# Combining powers of linkage and association mapping for precise dissection of QTL controlling resistance to gray leaf spot disease in maize (*Zea mays* L.)

**DOI:** 10.1186/s12864-015-2171-3

**Published:** 2015-11-10

**Authors:** Jafar Mammadov, Xiaochun Sun, Yanxin Gao, Cherie Ochsenfeld, Erica Bakker, Ruihua Ren, Jonathan Flora, Xiujuan Wang, Siva Kumpatla, David Meyer, Steve Thompson

**Affiliations:** Dow AgroSciences, 9330 Zionsville Road, Indianapolis, IN 46268 USA

**Keywords:** Genome-wide association mapping, *Zea mays*, Genetic linkage mapping, Gray leaf spot, Quantitative trait loci mapping, Disease resistance, High resolution power, High QTL detection power, Marker-assisted selection, Single nucleotide polymorphism

## Abstract

**Background:**

Gray Leaf Spot (GLS causal agents *Cercospora zeae-maydis* and *Cercospora zeina*) is one of the most important foliar diseases of maize in all areas where the crop is being cultivated. Although in the USA the situation with GLS severity is not as critical as in sub-Saharan Africa or Brazil, the evidence of climate change, increasing corn monoculture as well as the narrow genetic base of North American resistant germplasm can turn the disease into a serious threat to US corn production. The development of GLS resistant cultivars is one way to control the disease. In this study we combined the high QTL detection power of genetic linkage mapping with the high resolution power of genome-wide association study (GWAS) to precisely dissect QTL controlling GLS resistance and identify closely linked molecular markers for robust marker-assisted selection and trait introgression.

**Results:**

Using genetic linkage analysis with a small bi-parental mapping population, we identified four GLS resistance QTL on chromosomes 1, 6, 7, and 8, which were validated by GWAS. GWAS enabled us to dramatically increase the resolution within the confidence intervals of the above-mentioned QTL. Particularly, GWAS revealed that *QTLGLSchr8,* detected by genetic linkage mapping as a locus with major effect, was likely represented by two QTL with smaller effects. Conducted in parallel, GWAS of days-to-silking demonstrated the co-localization of flowering time QTL with GLS resistance QTL on chromosome 7 indicating that either QTLGLSchr7 is a flowering time QTL or it is a GLS resistance QTL that co-segregates with the latter. As a result, this genetic linkage – GWAS hybrid mapping system enabled us to identify one novel GLS resistance QTL (*QTLGLSchr8a)* and confirm with more refined positions four more previously mapped QTL (*QTLGLSchr1, QTLGLSchr6, QTLGLSchr7,* and *QTLGLSchr8b)*. Through the novel Single Donor vs. Elite Panel method we were able to identify within QTL confidence intervals SNP markers that would be suitable for marker-assisted selection of gray leaf spot resistant genotypes containing the above-mentioned GLS resistance QTL.

**Conclusion:**

The application of a genetic linkage – GWAS hybrid mapping system enabled us to dramatically increase the resolution within the confidence interval of GLS resistance QTL by-passing labor- and time-intensive fine mapping. This method appears to have a great potential to accelerate the pace of QTL mapping projects. It is universal and can be used in the QTL mapping projects in any crops.

**Electronic supplementary material:**

The online version of this article (doi:10.1186/s12864-015-2171-3) contains supplementary material, which is available to authorized users.

## Background

Gray leaf spot (GLS) is one of the most important foliar diseases of maize in all areas where the crop is being cultivated. Severity of GLS depends on climate conditions suitable for fungus development. Disease is prevalent in the areas where dewy mornings are followed by a hot humid afternoon and relatively cool nights. In the USA, damages caused by GLS had been mild up to the 1970s. However, the introduction of reduced tillage practice as a measure to fight soil erosion created favorable conditions for the pathogen to overwinter in the cornfield and re-infect plants in the summer [1]. As it was predicted in the early 80s, during the last 20 years the importance of GLS in the USA has increased [2]. Although in the USA the situation with GLS severity is not as critical as in sub-Saharan Africa or Brazil, the evidence of climate change, increasing corn monoculture as well as narrow North American resistant germplasm can turn the disease into a serious threat to US corn production. Two species of *Cercospora*, namely *C. zeae-maydis* [3] and *C. zeina* [4], cause GLS. In the Unites States *C. zeae-maydis* occurs everywhere where corn is being cultivated, whereas *C. zeina* is mainly found on the East coast [4]. However, despite the presence of two species of Cercospora, the specificity of GLS resistance to either species have not been observed implying that GLS resistance is effective against both C. *zeae-maydis* and *C. zeina* [5].

The development of GLS-resistant cultivars through conventional or molecular breeding is one way to control the disease and ensure the security of corn production in the USA. Conventional breeding of GLS resistant cultivars has been difficult due to the complexity of the trait. Although GLS resistance is a highly heritable trait [6–8], it is controlled by many minor quantitative trait loci (QTL) [9, 10]. In fact, within the last 20 years using various sources of resistance, types of mapping populations, molecular markers and environments, over 57 GLS resistance QTL were detected in all 10 chromosomes of maize [11–13], out of which 31 were bioinformatically claimed to be false-positive [14]. Molecular breeding is a promising tool to breed GLS resistant corn cultivars. However, its success heavily relies on the availability of molecular markers that are physically close to QTL controlling the resistance to the disease.

Despite the substantial number of GLS resistance QTL mapping efforts using bi-parental mapping populations, majority of studies have mostly reported molecular markers flanking QTL confidence intervals which represented large segments of chromosomes. In many cases these markers are very far from the causative mutations, easily lost during meiotic recombination, and consequently not useful in molecular breeding. One of the major reasons is the use of small bi-parental mapping populations with low genome resolution power. In recent GLS QTL mapping studies the sizes of bi-parental mapping populations ranged between 100–300 individuals [12, 15–17]. Although a bi-parental genetic mapping approach offers high QTL detection power, its resolution remains low due to inaccurate recombination information [18], which leads to a strong statistical association of QTL with the block of markers that physically span large chromosomal segments. To capture all possible recombination events, one can increase the size of the mapping populations, which is a very time- and cost- intensive procedure, especially when dealing with immortal populations such as recombinant inbred lines (RILs) or double haploids (DH). However, even fine mapping in many cases will not help to delimit a QTL interval to a significantly smaller segment of DNA because of a limited number of meiotic recombination events [19]. Another way to increase the resolution within a QTL confidence interval and discover additional recombination events was proposed to be the application of high-density marker technologies [20].

In contrast to the bi-parental approach, the linkage disequilibrium-based genome-wide association study (GWAS) overcomes the problems related to the lack of recombination events due to the structure of the association mapping population which is composed of genetically un-related individuals with unknown pedigrees and accumulates a larger number of historical recombination events that occurred in the past [21]. However, unlike the bi-parental approach of QTL mapping, the detection power of GWAS is fairly low and the method is prone to discover false-positive QTL [22].

In this study we combined the high QTL detection power of the bi-parental approach with the high resolution power of GWAS by applying a genetic linkage - GWAS hybrid mapping system to dissect QTL controlling GLS resistance and identify closely linked molecular markers for robust marker-assisted selection and trait introgression. Briefly, one small bi-parental mapping population and an Association Panel of 300 maize inbred lines, which also included the parents of the bi-parental population, were simultaneously tested in four environments (two years x two locations) for their reaction to *Cercospora* . Using the bi-parental mapping population, confidence intervals supporting GLS resistance QTL were identified. In parallel, GLS resistance QTL were also discovered by GWAS. Then the locations of GWAS-detected QTL were superimposed with QTL intervals identified by the bi-parental mapping approach. Single nucleotide polymorphism (SNP) markers residing within the confidence interval as defined through the bi-parental approach and associated with GLS resistance QTL as discovered by GWAS were further validated for their potential usefulness in marker-assisted selection (MAS).

## Methods

### Genetic Materials

Two mapping populations were used in this study. The DH population was developed from a cross between two Dow AgroSciences (DAS) proprietary maize inbred lines. One of the parents, DAS-001 (GLS resistant), is a temperate maize line of South American origin. The second parent, DAS-002 (GLS susceptible) is a temperate maize line of U.S. Corn Belt origin. The DH population was represented by 72 lines, which were assessed for the disease. This bi-parental population was used to conduct genetic linkage mapping of QTL controlling GLS resistance.

The second population, Association Panel, was developed to conduct GWAS. The Association Panel was comprised of 300 maize inbreds, including 215 DAS proprietary lines of North and South American origin, 27 ex-PVP lines, 37 CYMMIT lines, and 21 lines from the National Plant Germplasm system (Additional file 1). All lines in the Association Panel were chosen based on their previously known reaction to GLS and represented four major categories: GLS susceptible, moderately GLS susceptible, moderately GLS resistant, and GLS resistant. Software STRUCTURE [Version 2.3.4 (Jul 2012)] [23, 24] was used to infer the population structure of the Association Panel. Based on prior knowledge of this Association Panel, the range of the subpopulations tested in STRUCTURE was set from 1 to 5. The analysis was repeated five times with 100,000 Markov Chain Monte Carlo (MCMC) replicates and 100,000 burn-ins. The optimal number of clusters representing population substructure was determined by the Delta K [25], which was calculated based on the second-order rate of change in estimated log likelihood [LnP(D)] between successive values for K.

### Field trials

Both the bi-parental mapping population and Association Panel were planted in four environments: 2011 and 2012 in Davenport, IA (hereafter referred to as DAV-2011 and DAV-2012, respectively), and 2011 and 2012 in Mount Vernon, IN (hereafter referred to as MTV-2011 and MTV-2012). Fifteen kernels per line were planted per row within a 10 ft plot in each environment. Each block contained five replicates of each parents used as checks. All experimental plants and checks were artificially inoculated. Checks were used to insure the uniformity of artificial inoculation. GLS inoculum for the field studies was prepared as described in [26] with some modifications. Briefly, *Cercospora* spores, collected from heavily infested field grown corn plants, were grown in V8® juice liquid shake culture with a ten grams per liter base of carboxyl methyl cellulose (CMC, 90,000 MW). After seven to twelve days of growth, the liquid culture was diluted with water at a 1:1 ratio and blended to free spores from the balls of stroma, followed by addition of CMC at a ratio of five grams per liter of the suspension. The solution was filtered and resulted in a final dilution of inoculum diluted at a ratio of 1:3 with a final concentration of five grams per liter of CMC. CMC was used to stabilize the suspension and increase its adhesiveness to the leaf surface in a non-phytotoxic manner. Liquid inoculum was sprayed twice onto the whole plant with backpack sprayers. The first spray was at V8-9 stage, followed with 7–12 day interval around V11-12 stage. Both applications were sprayed in the evenings. To ensure successful epidemic, the first six plants of each plot were left unsprayed to contrast with the sprayed plants within the same plot. To ensure uniform coverage of the whole plant canopy, 60° cone sprayer tip was position roughly a 45° angle and 18–20 in. above the whorl leaf and each plot was sprayed by walking up and down at a constant speed from both sides of the plots. *Cercospora* inoculum was not characterized at molecular level to reveal the content of the fungal population.

Independent field trial was conducted in Sidney (IL) in 2012, where flowering time data were collected from 254 representatives of the Association Panel. Flowering time data were represented by days to silking (DTS) and measured as days from planting to silk emergence in 50 % of plants in row (Additional file 2).

### Disease rating

Entries in each environment were rated two to three times: immediately after 50 % of the plants in a row reached mid silk (female flowering) and three weeks after entire row reached mid silk. On average, phenotypic data were collected 39 and 60 days after the last inoculation. Depending on the type of GLS resistance, maize responds differently to the pathogen: rectangular necrotic lesions are characteristic of susceptible lines, flecks are indicative of resistance, while chlorotic lesions with orange or yellow borders/halo are characteristic of intermediate resistance [1]. Biological weight indices were assigned to each type of lesion: necrotic lesions – 0.75, chlorotic lesions – 0.20, and flecks – 0.05 so that the sum of those indices would be equal to 1. These indices were empirically deduced and developed from multiple previous field tests (data not shown). The second parameter taken into consideration was the percentage of infected area of a leaf covered by a predominant lesion type, rated on a 1 (3-9 % of infected leaf area) to 9 (>89 % of infected leaf area) scale. Lesion type and infection spread were measured on three leaves per plant: the leaf directly below the ear, the ear leaf and the leaf directly above the ear. To calculate the overall GLS severity of one plant per rating, the formula below was used:

GLS = [(LTIBE*PLSBE) + (LTIEL*PLSEL) + (LTIAE*PLSAE)]/3, where LTI is lesion type index, PLS – predominant lesion spread, BE, EL and AE – below ear leaf, ear level leaf and above ear leaf, respectively. Three plants per line were evaluated, and phenotypic data were averaged. The Area Under Disease Progress Curve (AUDPC) [27] was calculated. AUDPC was calculated across all environments and used as a trait for QTL mapping. The lower value of AUDPC corresponds with the more resistant phenotype.

### Statistical analyses

Pairwise Pearson correlation coefficients were calculated between mean AUDPC values at four environments: DAV-2011, DAV-2012, MTV-2011, and MTV-2012 using JMP software (version 10; SAS Institute, Gary NC). Mixed models were run using PROC MIXED in SAS (version 9.3; SAS Institute, Gary NC) with line, environment, and environment × line interaction as random effects. The significance levels of random effects were estimated with a type 3 F-test. Using the formula below, broad sense heritability was calculated based on a method described by Holland and Nyquist [28].

$$ H=\frac{\sigma_G^2}{\sigma_P^2}=\frac{\sigma_G^2}{\sigma_G^2+\frac{\sigma_{GE}^2}{e}+\frac{\sigma_e^2}{re}} $$

Where σ_G_^2^, σ_P_^2^, σ_GE_^2^ are the variances of genetic lines, phenotype, genetic by environment, respectively, σ_e_^2^ is residual variance, r is the number of replicates in each environment, and e is number of environments.

### Molecular markers and linkage map construction

DNA was extracted from eight leaf punches using the MagAttract 96 well DNA kit (QIAGEN, Hilden, Germany). Both mapping populations were genotyped by a custom iSelect [Infinium assay, Illumina (San Diego, CA)], which consisted of 33 K attempted bead types. The iSelect was composed of SNPs representing 27,494 maize genes (based on B73 RefGen_v2) in a ratio of one SNP per maize gene. As a result of genotyping, the DAS-001 x DAS-002 DH population revealed ~7200 polymorphic SNPs. Due to the small size of the mapping population and physical proximity of many SNPs, big clusters of co-segregating markers with the same genetic information were expected. To reduce the number of markers for genetic linkage analysis, several steps were undertaken after genetic linkage mapping was carried out. Polymorphic markers between the parental genomes were first clustered and initially ordered based on physical locations on the B73 reference genome (B73 RefGen_v2). Then the segregation patterns of all markers were explored. Based on the latest order, markers demonstrating the same segregating pattern as a neighboring marker were removed from further analysis. The initial genetic map for each chromosome was constructed using the greedy algorithm followed by a ripple function with a window size of six markers. Genetic distances were determined using the Haldane map function using functions available in “R\qtl” [29], a package in the “R” statistical analysis software [30]. The genetic map was further evaluated for small blocks of markers that had recombination patterns different than the flanking markers indicating that the markers were misplaced by the software. Blocks of three or less markers that had recombination fractions of 0.15 or greater than markers flanking the block were identified and removed. This iterative process was considered complete when no additional markers were removed based on duplicated marker patterns or unusual segregation patterns. Lastly, markers that were less than 0.2 centimorgan (cM) apart, were also removed from the genetic map. The final map of 1985 SNP markers, evenly distributed across ten maize chromosomes, was leveraged for QTL mapping.

### QTL mapping

#### Bi-parental approach

In this study, an extended composite interval mapping (ECIM) model [31] was used for QTL mapping. ECIM is similar to the composite interval mapping (CIM) model, which is the basis of the MapQTL software [32], as it expands the interval mapping (IM) model by including additional marker covariates. However, in contrast to CIM, the ECIM model increases the power of QTL detection through the inclusion of fixed experimental effects, such as location and year, into the analysis model. Particularly, in this study, the ECIM model allowed to incorporate data from all four environments into one analysis, which would be impossible to do using the traditional CIM model.

Due to the inclusion of marker and other fixed covariates within the ECIM model, the likelihood statistics utilized to obtain the LOD score fails to follow a predictable theoretical distribution. Therefore, the implementation of an empirical threshold to determine significance was not theoretically valid [33, 34]. As a solution, a novel bootstrap threshold algorithm was utilized, which provided accurate re-sampling to establish LOD score significance thresholds for the ECIM [31]. The bootstrap significance threshold algorithm is similar to the empirical threshold algorithm described by Churchill and Doerge [34] as both determine the maximum LOD score from a genome scan of the re-sampled data. However, the bootstrap threshold algorithm differs from the empirical permutation threshold algorithm as the former generates a new data set based on a bootstrap re-sampling of the centered residual effects. Residuals are generated by subtracting the parameter estimates for the additive, dominance, and covariate effects from the phenotype. The residuals are then centered at zero by subtracting their mean and these effects are resampled using a bootstrap algorithm. The bootstrap resampled phenotypes are obtained by summing covariate effect estimates to the resampled residuals to generate a null distribution of the phenotype. In the same process as the Empirical Threshold, LOD scores are calculated for each resampled data set for each marker. The maximum LOD score across the genome for each resampled data set is collected. From the realized distribution of maximum LOD scores the value of the maximum LOD score at the established percentile is utilized as the global LOD threshold [31]. In this study, the global LOD threshold was established at 3.23.

### Genome-wide association study

GWAS was conducted by internally developed SBayes method. Details of the method are described in Additional file 3. Briefly, GWAS implemented with this SBayes method consists of two steps as the statistical model underlying this method combines noise reduction and shrinkage of SNP effect components. To decrease noise in the marker data, supervised principal component analysis [35] was applied. The second step oversaw the actual GWAS performed using the Bayes-Cπ method [36] which fitted all markers simultaneously. In SBayes, the significance of SNP effects was measured by narrow sense heritability (h^2^), which was calculated by the formula below:

$$ {h_j}^2=\frac{\sigma_a^2}{\sigma_p^2}=\frac{2{p}_j\left(1-{p}_j\right){b}_j^2}{\sigma_p^2}, $$

where phenotypic variance *σ*_*p*_^2^ was estimated by the sample variance; additive variance was the function of SNP effect and allele frequency of *j*th SNP. Heritability as a measure of significance of a SNP effect in SBayes method is an equivalent of a *p*-value in traditional Q/K model. In order to calculate the significance of a SNP effect, the heritability values of individual chromosomes were computed first. Then heritability of every SNP within a chromosome was identified. The SNPs whose heritability values were higher than the threshold were considered as significant. In this study, the significance threshold was set to 0.3, indicating that only SNPs with at least 30 % of the maximum heritability of each chromosome would be picked as significant. In this study, the length of the support interval (window size) for each QTL position was set to be 7.5 cM from both sides of the SNP associated with the QTL, meaning that within this confidence interval (15 cM) the level of false positive rate is expected to be low. The SNP effect is the additive effect of the detected SNP. For any bi-allelic SNP [A/B], a positive effect suggests that the allele contributing to GLS severity comes from allele A and a negative effect suggests that the allele contributing to GLS severity comes from allele B. GWAS was conducted using ~25,000 SNP markers with minor allele frequencies (MAF) > 0.1.

### Delimiting QTL intervals based on GWAS

After the completion of genetic linkage mapping and GWAS, the physical positions of GWAS-detected markers were superimposed with the location of QTL confidence intervals identified by genetic linkage mapping. If GWAS detected marker was physically located under the QTL confidence interval, it was used as an anchor to delimit the physical span of QTL interval identified by genetic linkage analysis. A support interval for each QTL position defined by GWAS was 7.5 cM from both sides of the SNP markers associated with the GLS resistance QTL. In GWAS, support interval assumes that all markers located within that interval (in this study 15 cM) are significantly associated with QTL. Subsequently, the physical length of the support interval was calculated. Based on physical coordinates, GWAS-detected SNPs were first assigned to chromosomal bins at the public high resolution IBM2 2008 Neighbors map at Maize GDB (http://www.maizegdb.org/data_center/map). Using coordinates of public markers flanking a bin, physical (in base pair) and genetic (in cM) lengths of a bin were determined. Subsequently, the physical length of 1 cM of a bin was calculated. Left and right physical borders flanking GWAS detected SNP markers were calculated by the formula [physical position of a SNP +/− (7.5 cM * physical length of 1 cM of a chromosomal bin)].

### Single Donor vs. Elite Panel (SDvEP) method to discover markers suitable for marker-assisted selection (MAS) of GLS resistant maize lines

The main concept of the SDvEP method was described in [37]. Briefly, molecular markers, identified by genetic linkage mapping or GWAS as closely linked to a trait of interest are not always informative and accurate in MAS. Particularly, putative target alleles discriminated by those markers are not necessarily well conserved in genotypes carrying those traits, they can be also found in genotypes that do not possess the target trait. This might lead to selection of false positives during MAS. Using the SDvEP method one can mine for alleles within QTL support interval identified by genetic and/or association mapping which distinguish a donor of a trait of interest from a large number of lines that do not have that trait. One of the prerequisites of SDvEP is QTL mapping and the identification of the physical boundaries of the QTL support interval. The second step is the development of a panel of lines (hereafter referred to as Elite Panel) that do not have a target trait. To implement SDvEP, a single donor of a trait and entire Elite Panel should be genotyped. Genotyping can be done either using molecular markers or by sequencing. Whole genome sequencing would be ideal for SDvEP but it will be prohibitively expensive. Genotyping will be followed by mining alleles within QTL support intervals. The target alleles are those which discriminate the single donor of a trait from the members of the Elite Panel. In other words, SDvEP targets alleles that are conserved in the donor line only. Molecular markers developed based on those alleles can be claimed as suitable for MAS of a target trait.

In this study, the Elite Panel was represented by 109 maize inbred lines, which showed susceptibility to GLS across all environments. The representatives of the Elite panel were chosen based on the availability of necrotic lesions on the above the ear leaves, which indicated the lack of any resistance to the pathogen. Lines that were showing chlorotic lesions on the above the ear leaves were not included into the Elite Panel as they might contain certain level of resistance to GLS that allowed them to impede the disease spread. These lines were part of the Association Panel and also included the GLS susceptible parent of the DH population (DAS-002). The single donor of GLS resistance was represented by the DAS-001 line, one of the parents of the DH population. Furthermore, genotypic data of SNP markers located within the GLS resistance QTL support interval were compared between the single donor (DAS-001) and the Elite Panel. Several criteria were taken into consideration while evaluating markers for their usefulness in MAS: (1) a marker should be located within the QTL support interval identified by GWAS; (2) a marker should be polymorphic between the parents of the DH mapping population (DAS-001 and DAS-002) to enable MAS for GLS resistance coming from the DAS-001 background; (3) a GLS resistance allele discriminated by a marker should be conserved in the DAS-001 genetic background (as well as other GLS resistant maize lines) and absent in all GLS susceptible maize lines representing the Elite Panel. The latter criterion has a potential to reduce the risk of detecting false positive lines during MAS of GLS resistant lines. GLS resistant lines which showed no necrotic lesions on the leaves during the course of experiments were chosen to see whether putative GLS resistance alleles were also conserved in their genome. Twenty-three GLS resistant lines were randomly chosen for this panel.

## Results

### Phenotypic analysis

**Fig. 1:**
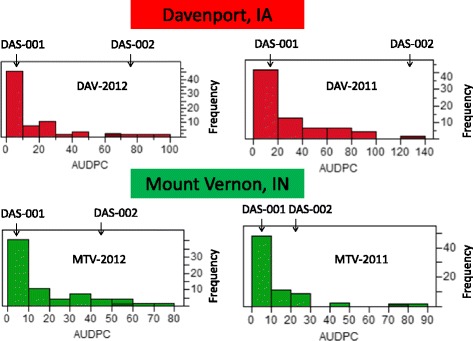
Distribution of GLS severity in the DAS-001 × DAS-002 DH mapping population. The x-axis represents the area under disease progress curve (AUDPC). The y-axis represents the number of individuals within a particular AUDPC category. Two vertical arrows represent the locations of the resistant (DAS-001) and susceptible (DAS-002) parents

DH lines representing a bi-parental mapping population and an entire Association Panel were evaluated for their reaction to *Cercospora* in four environments. As expected, the GLS resistant line DAS-001 showed a high level of resistance to the disease in all four environments (Fig. 1). The DAS-002 line was highly susceptible in DAV-2011 and DAV-2012 environments, while in MTV-2011 and MTV-2012 it showed moderate susceptibility to the disease due to hot and dry summers of 2011 and 2012 in Indiana, which did not favor the development of the infection (Fig. 1). Nevertheless, the pairwise Pearson correlation of the GLS Area Under Disease Progress Curve (AUDPC) in all four environments was highly significant (*P* < 0.0001) with correlation coefficients ranging between of 0.45-0.72 (Table 1) indicating that artificial inoculation was efficient to develop a biologically meaningful phenotype. In all four environments, the response of the DH population to disease pressure was continuously distributed, which suggested that GLS resistance is quantitatively inherited. Although disease severity distribution was continuous, it was L-shaped and skewed towards the resistant parent (Fig. 1). Resistance to GLS in both the DH population and the Association Panel was indicated to be controlled by genetic factors as broad sense heritability was 0.792 ± 0.044 and 0.804 ± 0.020, respectively. The variance components for all random-effect factors (environment, line, and environment-line interaction) for GLS severity were significantly different from zero (Table 2A, B). This analysis demonstrates that the genetics underlying GLS resistance substantially contributed to the overall phenotype as the variation assigned to the environment x line interaction was much smaller than the variation ascribed to lines.

**Table 1:** Pearson correlation coefficients between AUDPCs for GLS among four environments

Environment	DAV-2011	DAV-2012	MTV-201	MTV-2012
DAV-2011	1.00	*r* = 0.59	*r* = 0.68	*r* = 0.65
		(*P* < 0.0001)	(*P* < 0.0001)	(*P* < 0.0001)
DAV-2012	-	1.00	*r* = 0.45	*r* = 0.72
			(*P* < 0.0001)	(*P* < 0.0001)
MTV-2011	-	-	1.00	*r* = 0.60
				(*P* < 0.0001)
MTV-2012	-	-	-	1.00

**Table 2:** Analysis of variance component estimates (Var), standard errors (Std error), and P values of random effects in mixed models for GLS resistance across all environments for the DAS-001 x DAS-002 DH population (A) and Association Panel (B)

A.			
Random Effect	Var	Std Error	P value
Environment	57.23	59.09	<0.0001
Line	283.23	62.57	<0.0001
Line x Environment	154.84	26.01	<0.0001
Residual	186.74	10.77	
B.			
Random Effect	Var	Std Error	P value
Environment	331.35	247.24	<0.0001
Line	879.47	84.71	<0.0001
Line x Environment	379.72	33.59	<0.0001
Residual	481.61	14.24	

### Detection of GLS resistance QTL using a bi-parental approach

QTL mapping using a bi-parental approach resulted in the identification of four chromosomal landmarks associated with GLS resistance (Table 3). Three minor and one major QTL were detected on chromosomes 1, 6, 7, and 8, respectively. The QTL on chromosome 8 (*QTLGLSchr8*) explained ~26.5 % of the variation, while the QTL on chromosomes 1 (*QTLGLSchr1*), chromosome 6 (*QTLGLSchr6*), and chromosome 7 (*QTLGLSchr7*) were responsible for 4.55 %, 6.85 %, and 5.23 % of GLS resistance, respectively. In total, all four identified QTL explained 43.13 % of GLS resistance in the DAS-001 inbred line (Table 3).

**Table 3:** Gray leaf spot resistance quantitative trait loci detected by bi-parental approach using double haploid population

Name	Chr	Bins^a^	QTL confidence interval, bp		LOD^b^	R^2^	Additive Effect^c^
			Left border	Right border			
*QTLGLSchr1*	1	1.07-1.09	211,331,752	263,220,725	4.724933	0.045507	0.045507
*QTLGLSchr6*	6	6.01-6.04	26,909,746	120,538,443	6.9741	0.06859	32.11692
*QTLGLSchr7*	7	7.01-7.02	11,570,701	124,949,426	5.2492	0.052803	24.17346
*QTLGLSchr8*	8	8.02-8.04	14,069,105	118,971,618	22.83246	0.265049	105.1474

^a^Chromosomal bins were determined by the comparison of physical positions of markers flanking QTL confidence intervals and markers flanking bins in the high-resolution public map IBM2 2008 Neighbors at Maize GDB (http://www.maizegdb.org/data_center/map)

^b^Significant LOD threshold was set at 3.23

^c^Alleles contributed by the Susceptible (−) and Resistant (+) parent

To identify the physical span of the GLS resistance QTL, sequences of the SNP markers flanking the QTL confidence intervals were aligned against the maize B73 reference genome (version 2). Due to low resolution of the DH mapping population, all QTL intervals spanned large chromosomal regions. For example, *QTLGLSchr1* encompassed about a 50 Mb region on chromosome 1, while *QTLGLSchr7* harbored the longest region spanning almost 113 Mb (Table 3).

### Increasing the resolution within QTL intervals using a genome wide association study approach

Analysis of the structure of the Association Panel used in this study shows a sharp increase in the LnP(D) value for K = 3, indicating the presence of three possible clusters (Additional file 4). A similar pattern of clustering into three major subpopulations was supported by modified Rogers's distance phylogenetic tree (Additional file 5). The modified Roger’s distance estimates [38] were calculated from ~25000 SNP loci across genome. In the phylogenetic tree, three major sub-populations represented by stiff stalk, non-stiff stalk, and tropical maize inbred lines were observed (Additional file 5).

As the Association Panel included many GLS resistant lines with different genetic backgrounds (Additional file 1), it was expected that GWAS would identify many putative GLS resistance QTL. However, the focus of this study was those loci that fell within the confidence interval of GLS resistance QTL identified through the bi-parental approach. QTL located on chromosomes different from 1, 6, 7, and 8 as well as QTL which were detected on chromosomes 1, 6, 7, and 8 but were located outside of the confidence interval defined by genetic linkage analysis were not further considered. Those QTL were considered to be originated from a genetic background different from DAS-001.

On chromosomes 1, 6, 7, and 8, GWAS detected 13 GLS resistance QTL; 17 SNPs were associated with those QTL (Table 4). On chromosome 1, GWAS identified two SNP markers associated with a GLS resistance QTL, which were located in the 1.02 (QTL1.1) and 1.08 (QTL1.2) bins (Table 4). The position of QTL1.1 was outside of the confidence interval of *QTLGLSchr1* identified in the DH population (Table 3) and, therefore, was not a focus of this study. Meanwhile, the physical position of SNP marker PZE-101188909 associated with QTL1.2 fell within the *QTLGLSchr1* confidence interval (Tables 3 and 4) suggesting that QTL1.2 and *QTLGLSchr1* might represent the same GLS-resistance locus. Consequently, PZE-101188909 (Table 4) was used as an anchor marker to delimit the physical location of *QTLGLSchr1*. PZE-101188909 was located in bin 1.08 where the physical length of 1 cM was calculated to be 239,215 bp (Additional file 6). It was identified that the interval supporting QTL1.2 was about 1,794,113 bp (7.5 cM * 239,215 bp) from both side of the SNP marker. Consequently, the physical boundaries of *QTLGLSchr1* were delimited to a ~3.58 Mbp (232,515,087-236,103,313 bp) region within the 1.08 bin on maize chromosome 1 (Additional file 6), which was significantly reduced compared to the 52 Mb *QTLGLSchr1* interval identified by the bi-parental approach (Table 3). Seven flowering time (DTS) QTL were detected on chromosome 1. However, none of them were located within the confidence interval of *QTLGLSchr1* (Table 4).

**Table 4:** SNP markers associated with GLS resistance and days to silking (DTS) QTL identified by genome-wide association study

Chr	GWAS QTL	Bi-parental counterpart	Bins	Marker associated with GLS resistance QTL	Source of variation^a^	Minor allele frequency	Physical coordinate	SNP effects	Heritability of a SNP^b^	Maximum heritability of a chromosome
1	DTS_1.1		1.01-1.02	GLS-SNP-5365	[T/G]	0.30	12,255,366	0.07989852	0.001049039	0.003070336
1	GLS1.1		1.02	GLS-SNP-1590	[T/C]	0.43	15,354,490	−0.01960145	0.003070336	0.003070336
1	DTS_1.2		1.02	GLS-SNP-0156	[T/C]	0.42	22,510,157	0.04407421	0.003070336	0.003070336
1	DTS_1.3		1.03	GLS-SNP-0538	[A/G]	0.23	35,460,539	0.04052954	0.003070336	0.003070336
1	DTS_1.4		1.03	PZE-101061225	[G/T]	0.12	45,115,245	−0.04252824	0.003070336	0.003070336
1	DTS_1.5		1.03	GLS-SNP-2651	[A/G]	0.32	48,092,652	0.41783421	0.003070336	0.003070336
1	DTS_1.6		1.05	GLS-SNP-2138	[T/C]	0.46	101,832,139	0.43211445	0.003070336	0.003070336
1	DTS_1.7		1.07	GLS-SNP-5094	[A/G]	0.49	203,245,095	−0.69856342	0.003070336	0.003070336
1	GLS 1.2	*QTLGLSchr1*	1.08	PZE-101188909	[A/G]	0.23	234,309,200	−0.01785890	0.001971469	0.003070336
6	GLS 6.1	*QTLGLSchr6*	6.04	PZE-106058730	[A/G]	0.34	107,475,955	−0.00632726	0.000293801	0.00093559
6	GLS 6.2		6.04	PZE-106066216	[T/C]	0.42	118,304,229	0.01101481	0.000935590	0.00093559
6	DTS_6.1		6.05	Mo17-10397	[T/C]	0.41	151,787,329	0.04365824	0.000243429	0.00093559
6	GLS 6.3		6.05	PZE-106100504	[T/C]	0.32	153,414,853	0.00639998	0.000282006	0.00093559
6	DTS_6.2		6.06	GLS-SNP-4138	[A/C]	0.45	155,634,139	0.06046048	0.000701479	0.00093559
6	GLS 6.4		6.06	MAGI_11553-2	[T/G]	0.37	156,981,627	0.00610662	0.000281211	0.00093559
7	DTS_7.1		7.00	PZE-107000932	[T/C]	0.50	1,121,016	−0.00193525	0.000617793	0.000660398
7	GLS 7.1		7.00	PZE-107004786	[T/C]	0.40	3,074,900	0.00916059	0.000660398	0.000660398
7	GLS 7.2	*QTLGLSchr7*	7.02	PZE-107020739	[G/A]	0.37	19,500,572	−0.00603602	0.000265353	0.000660398
7	DTS_7.2		7.02	PZE-107020739	[G/A]	0.37	19,500,572	0.00248297	0.000107170	0.000660398
7	DTS_7.3		7.02	PZE-107083430	[C/T]	0.23	125,687,825	0.00226143	0.000585325	0.000660398
7	DTS_7.4		7.03	PZE-107094672	[A/G]	0.4	144,404,803	−0.00229674	0.000599634	0.000660398
7	DTS_7.5		7.03	PZE-107099659	[T/C]	0.32	154,472,118	0.00345513	0.002230712	0.000660398
8	GLS 8.1		8.01	GLS-SNP-7688	[A/G]	0.26	6,253,558	−0.00608691	0.000242320	0.000585845
8	GLS 8.2	*QTLGLSchr8a*	8.02	PZE-108020413	[T/C]	0.48	19,550,800	0.00468907	0.000177343	0.000585845
8			8.02	PZE-108022710	[T/G]	0.43	21,753,432	0.00584096	0.000269140	0.000585845
8	GLS 8.3	*QTLGLSchr8b*	8.03	PZE-108047250	[T/C]	0.46	79,142,282	−0.00521772	0.000218764	0.000585845
8			8.03	PZE-108050268	[A/T]	0.10	87,682,262	0.00806870	0.000204892	0.000585845
8	GLS 8.4		8.05-8.06	GLS-SNP-1344	[A/G]	0.30	154,247,344	−0.00922446	0.000585845	0.000585845
8			8.05-8.06	PZE-108098666	[T/C]	0.23	155,016,277	−0.00709668	0.000296117	0.000585845
8			8.05-8.06	PZE-108098682	[A/G]	0.22	155,029,958	−0.00890310	0.000450864	0.000585845
8	GLS 8.5		8.05-8.06	PZE-108101351	[A/C]	0.13	157,169,975	−0.01000713	0.000374213	0.000585845
8	DTS_7.1		8.05-8.06	GLS-SNP-8600	[T/G]	0.25	145,906,568	−0.00301422	0.000131115	0.000585845
8	DTS_7.2		8.06	PZE-108109056	[A/C]	0.31	162,535,081	0.00382457	0.000270955	0.000585845
8	DTS_7.3		8.06	GLS-SNP-6472	[T/C]	0.38	162,586,473	0.00290281	0.000154292	0.000585845
8	DTS_7.4		8.06	GLS-SNP-8795	[T/G]	0.45	163,616,966	−0.00209331	0.000435068	0.000585845

^a^Underlined allele is associated with GLS resistance

^b^In this study the significance threshold was set to 0.3, indicating that only SNPs with at least 30 % of the maximum heritability of each chromosome would be picked as significant

On chromosome 6, GWAS detected four GLS resistance QTL. Two of them were located in bin 6.04 (QTL6.1 and QTL6.2), while the other pair was located in bin 6.06 (QTL6.3 and QTL6.4) (Table 4). However, only QTL6.1 actually resided within the confidence interval of *QTLGLSchr6* identified in the DH mapping population, suggesting that both QTL6.1 and *QTLGLSchr6* represent the same GLS resistance locus. QTL6.1 was tagged by SNP marker PZE-106058730 (Tables 3 and 4). In bin 6.04, the physical length of 1 cM was estimated to be 244,961 bp (Additional file 6). Thus, the interval supporting the bin 6.04 QTL was about 1,837,208 bp (7.5 cM * 244,961 bp) from both side of the SNP marker. Consequently, the physical boundaries of *QTLGLSchr6* were delimited to a ~ 3.66 Mb (105,638,746-109,323,162) region within the 6.04 bin on maize chromosome 6 compared to the 83 Mb interval identified in the DH mapping population. Two DTS QTL were discovered on chromosome 6, and none of them were located within the confidence interval of *QTLGLSchr6* (Table 4)*.*

On the chromosome 7, GWAS discovered two QTL that resided in bins 7.00 and 7.02 and were designated as QTL7.1 and QTL7.2, respectively (Table 4). Out of two loci, QTL7.2 fell under the *QTLGLSchr7* interval identified in the DH mapping population (Tables 3 and 4). SNP marker PZE-107020739 associated with QTL7.2 served as an anchor landmark to delimit the confidence interval for *QTLGLSchr7*. In bin 7.02, a physical length of 1 cM was calculated to be 692,214 bp (Additional file 6). Subsequently, the QTL7.2 support interval was estimated to be 5,191,605 bp from both sides of the marker. Thus, physical boundaries of *QTLGLSchr7* were delimited to a ~ 10.3 Mb (14,308,967-24,692,177 bp) region within bin 7.02 on maize chromosome 7 and significantly reduced as compared to the ~113 Mb *QTLGLSchr7* interval identified in the DH mapping population. However, while mapping DTS QTL, the same marker, PZE-107020739 was associated with flowering time QTL, DTS_7.2 (Table 4). This finding indicates that *QTLGLSchr7* might be either flowering time QTL or this locus contains co-segregating GLS and DTS QTL.

In chromosome 8, GWAS revealed five GLS resistance QTL designated as QTL8.1, QTL8.2, QTL8.3, QTL8.4, and QTL8.5 (Table 4). QTL8.2 and QTL8.3 were located in bins 8.02 and 8.03, respectively, and within the *QTLGLSchr8* confidence interval. This finding suggests that *QTLGLSchr8,* which was thought to be a one major QTL based on genetic linkage analysis, most likely was represented by two QTL with lesser effects, further designated as *QTLGLSchr8a* (QTL8.2) and *QTLGLSchr8b* (QTL8.3) (Table 4). Two SNP markers, PZE-108020413 and PZE-108022710, are associated with *QTLGLSchr8a* (Table 4). Taking into account their physical positions as well as physical length of 1 cM in bin 8.02, the *QTLGLSchr8a* was estimated to span about a 6 Mb region (18,198,319-23,105,913) (Additional file 6). *QTLGLSchr8b* was calculated to span an approximately 19 Mb region within the 8.03 bin (73,871,364-92,953,180 bp) (Additional file 6). Thus, on chromosome 8 GWAS increased the resolution within the 104 Mb confidence interval of *QTLGLSchr8*, and allowed to dissect two loci with 6 and 19 Mb in length. No DTS QTL were identified within the *QTLGLSchr8a* and *QTLGLSchr8b* confidence intervals (Table 4).

### Discovery of SNP markers suitable for marker-assisted selection (MAS) of GLS resistance resulting from DAS-001 background

As GWAS was implemented with a limited number of SNPs (~25,000), not all polymorphisms existing between the members of the Association Panel were expected to be captured. Therefore, in this study, SNP markers that were associated with GLS resistance QTL were not considered in the context of putative causative mutations. However, as GWAS dramatically increased the resolution within GLS resistance QTL confidence intervals, further research was done to evaluate the usefulness of GWAS-detected SNP markers in MAS for GLS resistance resulting from a genetic background of DAS-001 and lines representing a similar source of resistance. Criteria to evaluate markers for their usefulness in MAS were described in the Methods section. SNP markers, which were associated with GLS resistance QTL based on GWAS were evaluated first. If GWAS-detected SNPs did not meet the criteria described in the Methods section, other SNPs within the support interval of *QTLGLSchr1, QTLGLSchr6, QTLGLSchr8a,* and *QTLGLSchr8b* were evaluated for their ability to track these QTL. No further actions were undertaken in this regards related to *QTLGLSchr7* as it was not clear whether this was a flowering time QTL or co-segregating with the former GLS resistance QTL. Any molecular marker linked to this locus would track both QTL and obscure the results of MAS.

The PZE-101188909 marker was revealed by GWAS to be associated with the *QTLGLSchr1* region. This SNP was also polymorphic between the parents of the DH mapping population. SDvEP analysis showed that the putative GLS resistance allele that was discriminated by the PZE-101188909 marker was well conserved in the DAS-001 genetic background and absent in the genetics of the 109 GLS susceptible lines (Additional file 7A). An interval of 3.59 Mb supporting *QTLGLSchr1* harbored 16 more markers that were polymorphic between the parents of the DH population. However, PZE-101188909 was the only SNP that fully met all criteria of a marker to track *QTLGLSchr1* region. Allele mining demonstrated that a putative GLS resistance allele discriminated by PZE-101188909 was present in 13 more GLS resistant maize lines representing the Association Panel (Additional file 7B).

The PZE-106058730 SNP was identified by GWAS as a marker associated with *QTLGLSchr6*. However, this marker was monomorphic among the parents of the DH population. The interval supporting *QTLGLSchr6* was further evaluated for the presence of informative markers. A DNA segment of ~ 3.67 Mb supporting *QTLGLSchr6* was landmarked by 59 SNP markers. However, only nine markers were polymorphic between DAS-001 and DAS-002. Unfortunately, none of the polymorphic markers clearly discriminated DAS-001 from the panel of GLS susceptible lines. The putative resistant alleles coming from DAS-001 genetics were also detected in many GLS susceptible lines. Thus, no informative marker was identified for *QTLGLSchr6*.

In the previous section, it was shown that two SNP markers, PZE-108020413 and PZE-108022710, were associated with *QTLGLSchr8a.* PZE-108020413 was monomorphic between the parents of the DH population, while PZE-108022710 was polymorphic between DAS-001 and DAS-002 but it did not meet SDvEP criterion. The segment of chromosome 8 that possessed *QTLGLSchr8a* (~4.9 Mb region) was landmarked by 80 markers, out of which 30 were polymorphic between DAS-001 and DAS-002. SDvEP revealed that only one SNP out of 30 polymorphic markers, PZE-108022834, clearly differentiated DAS-001 from the panel of susceptible lines (Additional file 7C). The PZE-108022834 marker was located only ~60 kb away from the GWAS-detected PZE-108022710 SNP. Thus, the PZE-108022834 marker was declared as a marker useful for tracking *QTLGLSchr8a*. This marker was also informative for 11 more GLS resistant maize inbred lines (Additional file 7D).

Two markers, PZE-108047250 and PZE-108050268, were identified to be associated with GLS resistance locus QTLGLSchr8b. However, only PZE-108050268 was polymorphic between DAS-001 and DAS-002. Moreover, the GLS resistant allele discriminated by this marker was conserved within DAS-001 genetic background and not revealed among GLS susceptible lines (Additional file 7E). Thus, PZE-108050268 was considered as a marker suitable for MAS of lines with *QTLGLSchr8b*. PZE-108050268 was also informative for nine more GLS resistant maize inbred lines (Additional file 7F). Information about MAS-suitable markers associated with GLS resistance is summarized in the Table 5.

**Table 5:** List of SNP markers associated with GLS resistance and informative in marker-assisted selection

QTL Name	SNP marker name	Sequence with the source of variation^a^	MAF^b^	Physical position
*QTLGLSchr1*	PZE-101188909	TTCAGGGTGCTGATTTATTTTTCCGGGCAGTAATCTCGAACATGGAAAAG[**A**/G]TTTATTTGAGCAGGAATCCTACAGCCAAAACCATTCTGGAGCTTGTACGA	0.23	234,309,200
*QTLGLSchr6*	none			
*QTLGLSchr8a*	PZE-108022834	CGCCGATGGATGGATAGACAGCAAATTCCGGTGAGCACATCGATCCGTTT[**T**/C]ATTCCATGCGCCGATCGATGCATATAGGTGCATGAAAACTTAATTACTCA	0.26	21,810,604
*QTLGLSchr8b*	PZE-108050268	AAAGCCAGCATACCAGTAGCACTAGTGAGTTAACCCCCCTGAAATTCTGC[A/**T**]GCAGCAGCAGTCTGGATCGCAGTCACCATGTCACCAGCCACCAGGGCATC	0.09	87,682,262

^a^Context sequence from which Infinium assay was designed. Single nucleotide polymorphism is shown in square brackets with putative GLS resistance allele underlined and bolded. Oligos for Infinium assay design were not included as they would always be *de novo* designed for any new order

^b^Minor allele frequency

## Discussion

### Phenotypic data

The two years of phenotypic data for the GLS resistance QTL mapping study (2011 and 2012) from Mount Vernon, IN and Davenport, IA had similar patterns: the humid and relatively hot summer of 2011 was followed by the extremely dry and hot summer of 2012. Based on the reaction of the GLS susceptible line, DAS-002, to *Cercospora* in 2011 and 2012, Davenport was a more favorable environment for disease development than Mount Vernon (Fig. 1). This can be explained by the geographical location of the lot where the DH mapping population and Association Panel were tested. In Davenport, the field is located within a valley, which accumulates a large amount of morning dew and creates favorable conditions for the development of the fungus. In 2012, due to severe drought and heat in Indiana, the development of *Cercospora* in Mount Vernon was suppressed (Fig. 1). However, despite the differences in weather conditions of 2011 and 2012, in all four environments the distribution of GLS resistance was continuous, L-shaped, and skewed towards the resistant parent (Fig. 1). The observed continuous distribution of GLS resistance indicates a quantitative nature of inheritance. However, the L-shaped, or gamma, distribution of GLS resistance in our experiments did not exactly align well with previously reported observations, where a normal distribution of this trait prevailed [7, 16, 39–41]. A gamma distribution of a trait controlled by QTL was previously reported in Drosophila [42] and cattle [43]. It is believed that gamma distributions are a characteristic of a trait that has many small effects QTL but few loci causing most of the genetic variation. On the other hand, a gamma distribution of GLS resistance in our experiment could be caused by the small size of the population [44] and relatively low disease pressure. In our study, heritability of GLS-resistance was calculated to be 0.792 ± 0.044, which was in correspondence with previous reports [16, 40].

While collecting phenotypic data, we observed the differences in disease manifestation among genotypes in terms of the predominant type of lesions affecting the leaves and the rate at which disease progressed vertically within a plant. To reflect those differences and conduct accurate phenotyping we developed a data collection methodology which would carry as much biological meaning as possible. Most GLS severity scoring methods take into consideration the area of a leaf affected by disease and assign scores from 1 (symptomless) to 9 (dead) [16, 40]. However, this disease rating does not take into consideration the type of predominant lesions that covers a leaf. For instance, if leaves of genotypes A and B are 50 % affected by elongated rectangular necrotic and chlorotic lesions, respectively, then the conventional disease rating method will assign “seven” to both genotypes. However, there is obvious biological difference between plants A and B as a rectangular necrotic lesion is an indicator of complete susceptibility as it produces a large amount of conidia, while a chlorotic lesion produces few conidia [45], which is an indicator of moderate resistance to GLS. To reflect this difference in our phenotyping methodology, we assigned weighted indices to each type of lesion (see Methods section). The rate at which GLS is spreading vertically across the plant was also taken into consideration. Even if two plants are affected by the same type of lesion, the rate at which those lesions spread towards the leaf above the ear indicates the difference in GLS resistance among those two plants. To address this component of the reaction of maize to GLS, we evaluated three leaves within a plant, namely, leaf below ear, ear leaf and leaf above ear, which gave us a very good picture of disease spread dynamics. Ear leaf and leaf above ear were also evaluated for GLS severity at Zwonitzer et al. [15].

### QTL mapping using combined bi-parental and GWAS approaches

With genetic linkage mapping we discovered four GLS resistance QTL, three QTL with minor effects (*QTLGLSchr1, QTLGLSchr6,* and *QTLGLSchr7*) and one major QTL (*QTLGLSchr8).* In our experiments, the confidence intervals of GLS resistance QTL discovered by genetic linkage analysis were large, which was expected taking into account the small size of the bi-parental mapping population. To increase the resolution within the confidence intervals, we implemented GWAS simultaneously with bi-parental QTL mapping. GWAS is known to have high resolution power due to historical recombination events accumulated within an association panel [46]. In this study, we took advantages of both QTL discovery methods, namely high detection power of the bi-parental approach and high resolution power of GWAS to robustly identify GLS resistance QTL. Remarkably, GWAS drastically increased the resolution within the confidence intervals of GLS resistance QTL. In case of *QTLGLSchr1, QTLGLSchr6,* and *QTLGLSchr7* the resolution was increased ~14, ~23 and ~10 fold, respectively. In addition, GWAS revealed that *QTLGLSchr8,* which was claimed as a locus with major effect based on genetic linkage mapping, was likely to consist of two QTL. Major QTL fractionation was also previously reported in maize and tomato [13, 47, 48]. Thus, the combination of bi-parental and GWAS approaches helped us to further refine the GLS resistance loci to an extent that it became possible to separate the effects of two co-segregating QTL (on chromosome 8), which normally is recommended to be carried out through the painfully long and expensive process of developing near isogenic lines (QTL-NILs) [13, 49]. However, we also have to state that this methodology is not designed to identify causative mutations underlying GLS resistance. Although GWAS is a great tool to identify molecular markers linked to QTL, the analysis is primarily based on molecular markers with higher minor allele frequencies as the removal of rare alleles is a choice to reduce the number of false-positive QTL [50]. However, studies showed that most phenotypic variations are due to rare alleles, suggesting the importance of these rare alleles in tagging biologically meaningful associations [51]. In order to further mine GLS resistance QTL intervals identified and refined in this study and discover causative mutations, fine mapping and gene cloning coupled with functional genomics studies are needed.

### Novel GLS resistance QTL

To confirm whether we identified novel GLS resistance QTL, comparative analysis of the physical positions of previously reported QTL with loci identified in this study was conducted. As GLS symptoms rapidly manifest during flowering time [16], we also carried out GWAS analysis of DTS to see whether any GLS QTL coincides with DTS QTL. Surprisingly, on chromosome 7 GLS resistance QTL (*QTLGLSchr7)* co-localizes with DTS QTL (Table 4). Based on this research it is still premature to claim that chromosome 7 does not contain GLS resistance QTL and instead it harbors flowering time QTL as both QTL might co-segregate. Several studies also mapped GLS resistance QTL to this region and did not find any co-localized flowering time QTL [13, 16], although they collected days-to-anthesis as a flowering time data while we used days to silking. Further fine mapping of the region is needed to confirm whether locus contains both GLS and DTS QTL or the latter only. Remaining four GLS resistance QTL, *QTLGLSchr1, QTLGLSchr6, QTLGLSchr8a, and QTLGLSchr8b* did not show any co-localization with DTS QTL.

Chromosome 1 appears to be one of the hubs of GLS resistance QTL as ten out of 13 bins on the chromosome have a QTL mapped by various researchers (Table 6). In our study, *QTLGLSchr1* is located in bin 1.08. The only QTL that has been mapped so far onto bin 1.08 was described by Zwonitzer *et al.* [15] (Table 6). Comparison of physical boundaries of the above-mentioned QTL and *QTLGLSchr1* confirmed that they did not overlap but were very close to each other (Table 6). In fact, the confidence interval of GLS resistance QTL discovered by Zwonitzer et al. [15] goes towards bin 1.09, while *QTLGLSchr1* resides at the distal portion of bin 1.08. Based on the unique physical position of *QTLGLSchr1* only, one can argue that this GLS resistance QTL is novel. However, taking into account that Zwonitzer *et al.* [15] mapped GLS resistance QTL using a small RIL population and have never done fine mapping, the position of their bin 1.08 QTL might not be as accurate as the location of *QTLGLSchr1*. Consequently, there is a probability that *QTLGLSchr1* is basically the same QTL that Zwonitzer *et al*. [15] discovered but with a more refined position.

**Table 6:** Comparison of locations of GLS resistance QTL identified in this and previous studies

Chr	Bin	Flanking markers (bi-parental approach)/a marker (GWAS)	Physical distance between flanking markers	Mapping method	Reference
1	1.01	SYN20881	11,914,709	GWAS	[52]
	1.02	PZB01957.1	22,892,866-28,421,841	NAM (joint linkage mapping)	[13]
	1.04	PHM5098.25	56,747,253–83,780,725	NAM (joint linkage mapping)	[13]
	1.04-1.06	asg30^a^-bnl5.59^a^	(60,090,292 – 60,678,227) - (183,804,477-183,817,286)	Bi-parental	[17]
	1.05	asg3^a^-umc1515^a^	(77,240,735-83,433,335) – (97,880,433-103,311,831)	Bi-parental	[16]
	1.05	PZE-101097594	90,945,315	GWAS	[52]
	1.05	PZE-101101408	97,337,186	GWAS	[52]
	1.05	bmc1811^b^	82,574,898- 175,642,920	Bi-parental	[41]
	1.05-1.06	PZA01041.1^c^-bnlg1057^a^	129,815,592 - (189,086,513 - 191,089,856)	Bi-parental	[15]
	1.06	CSU3^a^ –CSU61^a^	(82,574,898- 82,577,349) (180,716,274- 181,194,957)	Bi-parental	[39]
	1.06	CSU92^a^ -bnl5.59^a^	(183,804,477 - 183,817,286) – (183,804,477 - 183,817,286)	Bi-parental	[10]
	1.06	PHM1968.22	161,027,952–208,733,347	NAM (joint linkage mapping)	[13]
	1.07	bmc1025^b^	199,107,718-228,644,352	Bi-parental	[41]
	1.08	TIDP5276^c^ – Bz2-2^c^	232,515,087 - 236,103,313	Bi-parental/GWAS	This study
	1.08-1.09	Bz2.2^d^-PHM14475.7^c^	241,373,004 - 257,186,738	Bi-parental	[15]
	1.10	umc147b	280,774,747-282,021,034	Bi-parental	[12]
	1.09-1.11	bnlg1720^a^ -umc1500^a^	(274,709,266-283,188,769) - (283,188,067-287,148,444)	Bi-parental	[40]
6	6.00	PZE-106000325	702,334	GWAS	[52]
	6.02-6.04	PZA00214.1	86,257,528–113,885,960	NAM (joint linkage mapping)	[13]
	6.02-6.05	Npi1373^b^-umc46^a^	(71,014,931 – 97,108,931) - (143,466,865-144,383,248)	Bi-parental	[39]
	6.04-6.05	PZA02673.1	118,087,791–147,224,252	NAM (joint linkage mapping)	[13]
	6.04	Idp4869 d – umc1857^d^	105,638,745 - 109,323,163	Bi-parental/GWAS	This study
	6.05	PZE-106073523	129,373,812	GWAS	[52]
		SYN26162	146,548,071		
	6.06-6.07	Umc1423	153,804,114 – 169,184,492	Bi-parental	[12]
		Umc36^b^			
7	7.02	asg34^a^ – umc116^a^	(14,027,268-14,618,739)-(127,094,683-130,251,052)	Bi-parental	[39]
	7.02	Umc1393	118,052,274 – 120,761,437	Bi-parental	[12]
	7.02	TIDP5499^d^ – crt2^d^	14,308,967 - 24,692,177	Bi-parental/GWAS	This study
	7.02	bnlg398^a^ -bnlg657^a^	(21,547,438-23,542,864)-(129,109,884-129,237,926)	Bi-parental	[16]
	7.02	PZE-107040293	67,970,430	GWAS	[52]
		PZE-107040370	68,121,109		
	7.02	phm4818.15^c^ – pza00132.17^c^	31,773,571-79,009,472	Bi-parental	[15]
	7.02-7.03	PZA00986.1	13,174,365–142,783,202	NAM (joint linkage mapping)	[13]
	7.02-7.03	bnlg1808	129,908,091	Bi-parental	[12]
		bnl15.21	132,550,575		
	7.03	PZE-107086511	136,155,325	GWAS	[52]
		SYN38495	141,187,793		
	7.03	SYN34849	152,695,327	GWAS	[52]
	7.03-7.04	umc111(psy3)^a^ -asg32^a^	(143,407,361-147,088,644)-(157,471,090-158,238,561)	Bi-parental	[39]
	7.03	bmc1305^b^	129,865,901 - 156,132,738	Bi-parental	[41]
8	8.01	SYN10053	1,816,317	GWAS	[52]
	8.01	GZ204^e^-IDP5^e^	8,616,802 - 10,074,106	Bi-parental	[40]
	8.02	Umc1974^d^ -TIDP8777^d^	18,198,319 - 23,105,913	Bi-parental/GWAS	This study
	8.03	PZA01470.1	23,769,876–101,178,933	NAM (joint linkage mapping)	[13]
	8.03	PZE-108028005	28,557,135	GWAS	[52]
	8.03	IDP8925^d^ -TIDP2787^d^	73,871,364 - 92,953,180	Bi-parental/GWAS	This study
	8.05	PZE-108075552	129,767,067	GWAS	[52]
	8.05	ufg80^a^ -bnlg666^a^	(130,740,118-131,241,328)-(133,561,516-133,936,736)	Bi-parental	[16]
	8.05	umc89^a^ -csu31^a^	(135,953,318-136,041,040)-(142,142,542-160,300,237)	Bi-parental	[39]
	8.06	PZA03651.1	135,091,499–156,907,035	NAM (joint linkage mapping)	[13]
	8.06	PZE-108108866	160,936,029	GWAS	[52]
	8.06	umc117^a^ -umc216(ald2)^a^	(162,531,179-162,630,591)-(163,307,256-163,309,969)	Bi-parental	[10]
	8.08	Dupssr14^e f^-phm14046.9^f^	(171,763,860-171,763,964)-175,362,738	Bi-parental	[15]

^a^Precise physical position of a marker was not provided at IBM2 2008 Neighbors genetic map. Instead, MaizeGDB suggests chromosomal interval where this marker could be located (http://www.maizegdb.org/data_center/map)

^b^Physical position of the marker was impossible to identify as the sequence information of the marker used in the study was not provided by authors. Instead, the physical borders of a bin where marker was reported to be located were provided

^c^Physical position of the marker was precisely identified based on sequence information of the context sequence leveraged from http://www.panzea.org/#!data/cyci

^d^Physical boundaries of QTL identified in this study were shown by public markers whose positions were very close to proprietary markers used to map GLS resistance QTL in this study

^e^Physical position of the markers were determined by the aligning sequences of PCR primers of flanking markers provided in the paper

^f^Physical position of the marker was determined by the aligning its sequences of PCR primers provided at Maize GDB

*QTLGLSchr6,* mapped to the chromosome 6 bin 6.04, does not appear to be novel GLS resistance QTL as its position overlaps with previously reported QTL on chromosomes 6 [13, 39] (Table 6). However, compared to GLS resistance QTL described by Clements *et al*. [39] and Benson *et al.* [13], *QTLGLSchr6* was delimited to much smaller segments of the chromosome 6 .

Chromosome 8 is another harbor for GLS resistance QTL as they were previously mapped to five out of eight bins (Table 6). The majority of studies mapped GLS resistance QTL to chromosomal bins 8.05 and 8.06, which span a 130–175 Mbp region on chromosome 8 [10, 12, 15, 16, 39, 41] (Table 6). Recently, a research group from the National Maize Improvement Center of China discovered major GLS resistance QTL on chromosome 8. Their initial mapping efforts using a small population resulted in the discovery of GLS resistance QTL in bins 8.01-8.03 (three environments) and 8.02-8.05 (one environment) [40]. However, Zhang *et al.* [40] declared that the location of GLS resistance QTL in 8.02-8.05 was possibly wrong due to inaccurate phenotyping. They went further and implemented fine mapping. As a result, they narrowed down the location of GLS resistance QTL on chromosome 8 to a 1.4 Mb interval, which spanned a region spanning 8,616,802-10,074,106 bp (bin 8.01). Interestingly, with the bi-parental mapping approach, we also mapped *QTLGLSchr8* (Table 3) to the bins 8.02-8.04, which was consistent with Zhang *et al.* [40]. However, our combined genetic linkage and GWAS efforts suggested the presence of two rather than one major QTL, *QTLGLSchr8a* and *QTLGLSchr8b,* in the region encompassing bins 8.02-8.04. Based on GWAS, *QTLGLSchr8a* and *QTLGLSchr8b* were mapped to bins 8.02 and 8.03, respectively. Recently Benson et al. [13] reported new GLS resistance QTL mapped to 8.03 bin too (Table 6). No GLS resistance QTL has been previously reported to be mapped to the bin 8.02, which suggests that *QTLGLSchr8a* might be a novel locus controlling GLS resistance.

### SDvEP a novel method to discover molecular markers effective in MAS

In GWAS experiments, molecular markers significantly associated with QTL are suggested to be closely linked to causative mutations or gene candidates [52–54]. Also, they were claimed as excellent tools for MAS [52–54]. In this study we observed that molecular markers significantly associated with GLS resistance QTL not always discriminated alleles that were well conserved in GLS resistant lines and absent in susceptible germplasm. In fact, putative GLS resistant alleles discriminated by SNP markers, associated with *QTLGLSchr6* and *QTLGLSchr8b,* were observed in susceptible genotypes too. This could be partially attributed to the fact that we carried out GWAS with a panel of several thousand generic SNPs that did not represent all polymorphisms available among the lines of Association Panel. A second reason could be that we discarded rare SNPs with minor allele frequencies <0.1 to enable GWAS. In human genetics the rare variants were proven to play an important role in controlling complex traits [55]. Although the purpose of this paper was not to discover the causative mutations controlling GLS resistance, we applied a novel method, SDvEP, to identify structural mutations, particularly SNPs, within GLS resistance QTL support interval that were well conserved in the donor of the trait (DAS-001 line) and absent in a large number of samples that do not have that trait. SNP markers discriminating those structural mutations would be considered suitable for MAS of GLS resistant lines. Using SDvEP we tested all SNP markers, including those with <0.1 MAF, located within a QTL confidence interval. SDvEP demonstrated that out of six markers linked to *QTLGLSchr1, QTLGLSchr6, QTLGLSchr8a,* and *QTLGLSchr8b* (Table 4) only two (PZE-101188909 linked to *QTLGLSchr1* and PZE-108050268 linked to *QTLGLSchr8a*) were proven to be suitable for MAS. The remaining four did not pass SDvEP as the putative resistant alleles discriminated by these markers were also present in the genetic background of other GLS susceptible lines. However, the SDvEP method helped us to find a target marker within the intervals supporting *QTLGLSchr8a* (Table 5). Interestingly, this SNP had <0.1 MAF (Table 5). Finally, no markers suitable for MAS of *QTLGLSchr6* were found. In this study, we did not further search for a MAS-suitable marker for *QTLGLSchr6*. However, this can be done by deep sequencing of the *QTLGLSchr6* support interval in DAS-001 GLS resistant line and the panel of 109 GLS susceptible lines followed by allele mining. In general, SDvEP coupled with deep sequencing can be a very powerful tool in finding MAS-suitable markers for the traits which are controlled by a single gene or by major QTL and several minor QTL.

## Conclusions

The application of a genetic linkage – GWAS hybrid mapping system enabled us to dramatically increase the resolution within the confidence interval of GLS resistance QTL, by-passing labor- and time-intensive fine mapping. This method appears to have great potential to accelerate the pace of QTL mapping projects. It is universal and can be applied for the dissection of any quantitatively inherited trait. Despite a large number of previously reported GLS resistance QTL, with the genetic linkage – GWAS hybrid mapping system we managed to identify one novel QTL controlling resistance to the disease, which most likely happened due to increased resolution within the QTL confidence interval offered by the method. Allele mining demonstrated that not all markers linked to the trait of interest can be implemented in MAS as putative GLS resistance alleles discriminated by those markers were also observed in GLS susceptible lines. In this study, we applied the novel SDvEP method for discovery of molecular markers within QTL support intervals that would be informative in MAS. This was done with the assumption that all polymorphisms within a QTL support interval were linked and provided the same genetic information. MAS-suitable markers were not considered as landmarks discriminating causative mutations as limited number of SNPs (~25,000) did not capture all polymorphisms available among the members of the Association Panel. However, the fact that putative resistance alleles discriminated by MAS-suitable markers were well conserved among GLS resistant maize inbred lines of diverse origin and were absent in susceptible genetic backgrounds could be an indicator that these markers were very close to causative mutations underlying GLS resistance.

### Availability of supporting data

The data sets supporting the results of this article are included within this article and its additional files.

## Additional files

Additional file 1:
**Maize inbred lines representing Association Panel used for genome wide association study with their reaction to** ***Cercospora*** **represented by the average value of the Area Under Disease Progress Curve (AUDPC) in all four environments.** (XLSX 23 kb) Additional file 2:
**Days to silking data collected from the representatives of the Association panel planted in Sidney (IL) in 2012.** Independent field trial was conducted in Sidney (IL) in 2012, where flowering time data were collected from 254 representatives of the Association Panel. Flowering time data were represented by days to silking (DTS) and measured as days from planting to silk emergence in 50 % of plants in row. (XLSX 14 kb) Additional file 3:
**Description of the development of SBayes method to carry out Genome-wide Association Study.** (DOCX 153 kb) Additional file 4:
**Analysis of the population structure within the set of 300 maize inbred lines used for the GWAS study.** Estimated LnP(D) and Delta K averaged over five repeats of STRUCTURE analysis. (DOCX 67 kb) Additional file 5:
**Phylogenetic tree of maize accessions in Association Panel.** Neighbor-joining tree of 300 maize accessions representing Association Panel. Three subgroups (stiff stalk, non-stiff stalk, and tropical germplasm) identified from the tree were color-coded. (PPTX 108 kb) Additional file 6:
**Physical boundaries of intervals supporting GWAS-detected gray leaf spot resistance QTL.** Using sequence information of GWAS-detected SNP markers associated with GLS resistance QTL and the public IBM2 2008 Neighbors map (http://www.maizegdb.org/data_center/map), corresponding chromosomal bins were identified. Physical and genetic lengths of a chromosomal bin were calculated by subtracting the physical (bp) and genetic (cM) coordinates of public markers flanking a bin where GLS resistance QTL resided. As not every flanking marker had a physical position in the public map, the closest marker with known physical positions was designated as the flanking marker. Support intervals for GWAS detected QTL were identified to be 7.5 cM from each side of a marker. Physical length of a QTL support interval = (physical length of a bin) /(genetic length of a bin)*7.5 cM. Physical boundaries of a QTL support interval = physical position of a SNP marker associated with GWAS detected QTL ± physical length of a QTL support interval. (DOCX 22 kb) Additional file 7:
**Marker-assisted selection-suitable markers identified by SDvEP method. The file contains several tabs.** Each tab is dedicated to one of the MAS-suitable markers. Each tab contains two images. The image on the left shows how a putative GLS resistance allele discriminated by a MAS-suitable marker is well-conserved in DAS-001 (GLS resistant parent of DH population) and absent in 109 GLS susceptible lines. The image on the right shows that a putative GLS resistance allele conserved in DAS-001 was also found in several other GLS resistance lines with a genetic background different from DAS-001. (XLSX 114 kb)

## Abbreviations

GLS: Gray leaf spot
GWAS: Genome-wide association study
QTL: Quantitative trait locus/loci
SNP: Single nucleotide polymorphism
MAS: Marker-assisted selection
ECIM: Extended composite interval mapping
CIM: Composite interval mapping
SDvEP: Single donor vs. elite panel

## References

[1] Ward JM, Stromberg EL, Nowell DC, Nutter FW (1999). Gray leaf spot: a disease of global importance in maize production. Plant disease.

[2] Latterell F, Rossi A (1983). Gray leaf spot of corn: a disease on the move. Plant Disease.

[3] Tehon L, Daniels E (1925). Notes on the parasitic fungi of Illinois: II. Mycologia.

[4] Wang J, Levy M, Dunkle LD (1998). Sibling species of Cercospora associated with gray leaf spot of maize. Phytopathology.

[5] Carson M, Goodman M, Williamson S (2002). Variation in aggressiveness among isolates of Cercospora from maize as a potential cause of genotype-environment interaction in gray leaf spot trials. Plant Disease.

[6] Gevers H, Lake J, Hohls T (1994). Diallel cross analysis of resistance to gray leaf spot in maize. Plant disease.

[7] Gordon SG, Lipps PE, Pratt RC (2006). Heritability and components of resistance to Cercospora zeae-maydis derived from maize inbred VO613Y. Phytopathology.

[8] Coates S, White D (1998). Inheritance of resistance to gray leaf spot in crosses involving selected resistant inbred lines of corn. Phytopathology.

[9] Bubeck D, Goodman M, Beavis W, Grant D (1993). Quantitative trait loci controlling resistance to gray leaf spot in maize. Crop Sci.

[10] Maroof MS, Yue Y, Xiang Z, Stromberg E, Rufener G (1996). Identification of quantitative trait loci controlling resistance to gray leaf spot disease in maize. Theor Appl Genet.

[11] Wisser RJ, Balint-Kurti PJ, Nelson RJ (2006). The genetic architecture of disease resistance in maize: a synthesis of published studies. Phytopathology.

[12] Berger DK, Carstens M, Korsman JN, Middleton F, Kloppers FJ, Tongoona P, et al. Mapping QTL conferring resistance in maize to gray leaf spot disease caused by Cercospora zeina. BMC genetics. 2014;15(1):60. 10.1186/1471-2156-15-60 PMC4059882 24885661

[13] Benson JM, Poland JA, Benson BM, Stromberg EL, Nelson RJ. Resistance to gray leaf spot of maize: genetic architecture and mechanisms elucidated through nested association mapping and near-isogenic line analysis. PLoS genetics. 2015;11(3):e1005045–5. 10.1371/journal.pgen.1005045 PMC4357430 25764179

[14] Shi L-Y, Li X-H, Hao Z-F, Xie C-X, Ji H-L, Lü X-L, Pan G-T ZHANGS (2007). Comparative QTL mapping of resistance to gray leaf spot in maize based on bioinformatics. Agricultural Sciences in China.

[15] Zwonitzer JC, Coles ND, Krakowsky MD, Arellano C, Holland JB, McMullen MD, Pratt RC, Balint-Kurti PJ (2010). Mapping resistance quantitative trait Loci for three foliar diseases in a maize recombinant inbred line population-evidence for multiple disease resistance?. Phytopathology.

[16] Balint-Kurti PJ, Wisser R, Zwonitzer JC (2008). Use of an advanced intercross line population for precise mapping of quantitative trait loci for gray leaf spot resistance in maize. Crop science.

[17] Lehmensiek A, Esterhuizen A, Van Staden D, Nelson S, Retief A (2001). Genetic mapping of gray leaf spot (GLS) resistance genes in maize. Theor Appl Genet.

[18] Bennewitz J, Reinsch N, Kalm E (2002). Improved confidence intervals in quantitative trait loci mapping by permutation bootstrapping. Genetics.

[19] Myles S, Peiffer J, Brown PJ, Ersoz ES, Zhang Z, Costich DE, Buckler ES (2009). Association mapping: critical considerations shift from genotyping to experimental design. The Plant cell.

[20] Pan Q, Ali F, Yang X, Li J, Yan J (2012). Exploring the Genetic Characteristics of Two Recombinant Inbred Line Populations via High-Density SNP Markers in Maize. Plos One.

[21] Nordborg M, Tavaré S (2002). Linkage disequilibrium: what history has to tell us. Trends Genet.

[22] Aranzana MJ, Kim S, Zhao K, Bakker E, Horton M, Jakob K, Lister C, Molitor J, Shindo C, Tang C (2005). Genome-wide association mapping in Arabidopsis identifies previously known flowering time and pathogen resistance genes. PLoS Genet.

[23] Pritchard JK, Stephens M, Donnelly P (2000). Inference of population structure using multilocus genotype data. Genetics.

[24] Falush D, Stephens M, Pritchard JK (2007). Inference of population structure using multilocus genotype data: dominant markers and null alleles. Mol ecol notes.

[25] Evanno G, Regnaut S, Goudet J (2005). Detecting the number of clusters of individuals using the software STRUCTURE: a simulation study. Mol ecol.

[26] Coates S, White D (1994). Sources of resistance to gray leaf spot of corn. Plant disease.

[27] Madden LV, Hughes G, Van den Bosch F (2007). The study of plant disease epidemics.

[28] Holland JB, Nyquist WE, Cervantes-Martínez CT (2003). Estimating and interpreting heritability for plant breeding: An update. Plant breeding reviews.

[29] Broman KW, Wu H, Sen S, Churchill GA (2003). R/qtl: QTL mapping in experimental crosses. Bioinformatics.

[30] R: A Language and Environment for Statistical Computing. http://www.R-project.org/, Accessed 4 November 2015.

[31] Ochsenfeld CA. Mixed models in quantitative rait loci and association mapping with bootstrap thresholds. http://docs.lib.purdue.edu/dissertations/AAI3379702/, Accessed 04 November 2015.

[32] Van Ooijen J (2004). MapQTL® 5, Software for the mapping of quantitative trait loci in experimental populations.

[33] Churchill GA, Doerge RW (2008). Naive application of permutation testing leads to inflated Type I error rates. Genetics.

[34] Churchill GA, Doerge RW (1994). Empirical threshold values for quantitative trait mapping. Genetics.

[35] Bair E, Hastie T, Paul D, Tibshirani R. Prediction by supervised principal components. J Am Stat Assoc 2006;101:(473): doi:10.1198/016214505000000628.

[36] Habier D, Fernando RL, Kizilkaya K, Garrick DJ (2011). Extension of the Bayesian alphabet for genomic selection. BMC bioinformatics.

[37] Mammadov J, Kumpatla SP (2013). Single donor versus elite panel methodology for identification of marker-assisted breeding friendly markers.

[38] Wright S. Evolution and the genetics of populations, volume 3: experimental results and evolutionary deductions, vol. 3: University of Chicago press; 1984.

[39] Clements MJ, Dudley J, White D (2000). Quantitative trait loci associated with resistance to gray leaf spot of corn. Phytopathology.

[40] Zhang Y, Xu L, Fan X, Tan J, Chen W, Xu M (2012). QTL mapping of resistance to gray leaf spot in maize. Theor Appl Genet.

[41] Pozar G, Butruille D, Silva HD, McCuddin ZP, Penna JCV (2009). Mapping and validation of quantitative trait loci for resistance to Cercospora zeae-maydis infection in tropical maize (Zea mays L.). Theor Appl Genet.

[42] Mackay TF (1996). The nature of quantittative genetic variation revisited: Lessons from Drosophila bristles. BioEssays.

[43] Hayes B, Goddard ME (2001). The distribution of the effects of genes affecting quantitative traits in livestock. Genet Sel Evol.

[44] Bost B, de Vienne D, Moreau L, Dillmann C (2001). Genetic and nongenetic bases for the L-shaped distribution of quantitative trait loci effects. Genetics.

[45] Freppon J, Pratt R, Lipps P (1996). Chlorotic lesion response of maize to Cercospora zeaemaydis and its effect on gray leaf spot disease. Phytopathology.

[46] Tian F, Bradbury PJ, Brown PJ, Hung H, Sun Q, Flint-Garcia S, Rocheford TR, McMullen MD, Holland JB, Buckler ES (2011). Genome-wide association study of leaf architecture in the maize nested association mapping population. Nature genetics.

[47] Studer AJ, Doebley JF (2011). Do large effect QTL fractionate? a case study at the maize domestication QTL teosinte branched1. Genetics.

[48] Johnson EB, Haggard JE, Clair DAS (2012). Fractionation, stability, and isolate-specificity of QTL for resistance to Phytophthora infestans in cultivated tomato (Solanum lycopersicum). G3: Genes| Genomes|Genetics.

[49] Kearsey M, Farquhar A (1998). QTL analysis in plants; where are we now?. Heredity.

[50] Abdurakhmonov IY, Abdukarimov A. Application of association mapping to understanding the genetic diversity of plant germplasm resources. Int J Plant Genomics. 2008;2008. 10.1155/2008/574927 PMC2423417 18551188

[51] Stich B, Melchinger A (2010). An introduction to association mapping in plants. CAB Reviews: Perspectives in Agriculture, Veterinary Science, Nutrition and Natural Resources.

[52] Shi L, Lv X, Weng J, Zhu H, Liu C, Hao Z, Zhou Y, Zhang D, Li M, Ci X (2014). Genetic characterization and linkage disequilibrium mapping of resistance to gray leaf spot in maize (Zea mays L.). The Crop Journal.

[53] Poland JA, Bradbury PJ, Buckler ES, Nelson RJ (2011). Genome-wide nested association mapping of quantitative resistance to northern leaf blight in maize. Proc Natl Acad Sci.

[54] Kump KL, Bradbury PJ, Wisser RJ, Buckler ES, Belcher AR, Oropeza-Rosas MA, Zwonitzer JC, Kresovich S, McMullen MD, Ware D (2011). Genome-wide association study of quantitative resistance to southern leaf blight in the maize nested association mapping population. Nat genet.

[55] Panoutsopoulou K, Tachmazidou I, Zeggini E (2013). In search of low-frequency and rare variants affecting complex traits. Hum Mol Genet.

