# Supplementary material for: Combining powers of linkage and association mapping for precise dissection of QTL controlling resistance to gray leaf spot disease in maize (Zea mays L.)
Source: BMC Genomics. 2015 Nov 10;16:916. doi: 10.1186/s12864-015-2171-3 (PMC4641357; doi:10.1186/s12864-015-2171-3)
Supplement: Additional file 3: — Description of the development of SBayes method to carry out Genome-wide Association Study. (DOCX 153 kb) [file 12864_2015_2171_MOESM3_ESM.docx]

**Additional file 3: Description of the development of SBayes method to carry out Genome-wide Association Study.**

# Method Description

SBayes method incorporates three major features, including (1) noise reduction component, (2) nonparametric regression (*i.e.* smoothing spline ANOVA), and (3) simultaneous marker fitting component. In SBayes, noise reduction in the marker data was achieved by the application of supervised principal component analysis (SPCA) [[1](#_ENREF_1)]. SPCA included the following steps:

1. Computing the regression coefficient for each marker on a single marker basis
2. Ranking markers by the absolute value of their regression coefficients and selecting a defined number of the top ranked markers to form a marker subset (MS) to construct the reduced data matrix
3. Performing PCA using the reduced data matrix to generate principal components (PC), referred further to as supervised principal components (SPCs).
4. SPCs explaining certain proportion of the data matrix variance were then considered as independent variables to fit a smoothing spline ANOVA model in reproducing kernel Hilbert spaces [[2](#_ENREF_2)] to adjust the original response.

The optimal number of markers in step 2 and the optimal PC variation in step 4 were determined separately by data-driven generalized cross-validation (GCV). In SBayes, simultaneous marker fitting was achieved by using the Bayes-Cπ method. The underlying model for Bayes-Cπ method is described below [[3](#_ENREF_3)]:

$$\hat{y}_{i}=\sum_{k=1}^{K} w_{ik}g_{k}+\sum_{j=1}^{P} x_{ij}\beta_{j}+\varepsilon_{i},$$

where g is the vector of fixed effects (e.g. mean and structure effects);$x_{ij}$is the ordinal-valued covariate (-1/0/1) for *j*th marker out of p total markers for *i*th individual;$\beta_{j}$is the regression coefficient; and$\varepsilon{}_{i}$ is the normally distributed model residual. Bayes-Cπ method was selected as it assumes a finite loci model as the underlying genetic architecture. Instead of assigning different variance to different marker loci, Bayes-Cπ assigns a common variance to all markers but incorporates the indicator function$\delta_{j}\left\{0,1\right\}$for each marker. The prior for variance components $\left(\sigma_{\beta}^{2}\text{ and }\sigma_{e}^{2}\right)$ is a scaled, inverse Chi-square distribution, with *S^2^* being scale parameter and *v* being degree of freedom [[4](#_ENREF_4)]. A gamma distribution was assigned to the hyperparameter *S^2^*. Bayes-Cπ was coded in C++ with$S_{\beta}^{2}$ = 4.23, $v_{\beta}$= 0.05 and$S_{e}^{2}$ =1,$v_{e}$ = 1. And smoothing spline ANOVA model was implemented using function “ssanova” in R package “gss”. Default arguments of *ssanova* function were used, *i.e.* argument “*method*” was set to “*v*” to let smoothness parameter *λ* be selected by GCV and “*type*” was set to “*cubic*” to use a cubic smoothing spline. In SBayes and Bayes-Cπ, SNP effects were tested for significance by calculating the narrow sense heritability, which is formulated as

$h_{j}^{2}=\frac{\sigma_{a}^{2}}{\sigma_{p}^{2}}=\frac{2p_{j}(1-p_{j})b_{j}^{2}}{\sigma_{p}^{2}}$ ,

where $\sigma_{p}^{2}$was estimated by the sample variance, and$p_{j}$was allele frequency of jth SNP. The maximum heritability was recorded for each chromosome and the threshold for each chromosome was set to be a series of numbers, *i.e.* 30%, 40%, 50%, 60%, 70%, and 80% of the maximum heritability. The SNPs with the values of heritability higher than the threshold were considered as significant.

Traditional QK method [[7](#_ENREF_7)] is based on mixed linear model:

where *b* is the fixed SNP effect, v is a vector of population effects, and *u* is the polygenic effect. Kinship was calculated according to [[8](#_ENREF_8)]. Mixed model was solved by R package “asreml-R”. The p-values of SNP effects were calculated from F-test and the false positive rate (FPR) was controlled by using R package “qvalue” to calculate q-values from the p-values. And SNPs whose q-values were smaller than the FPR were considered as significant.

# Simulation test to compare SBayes with QK and Bayes-Cπ methods

## Sumulation test design

Simulation was used to test and compare SBayes method with QK and Bayes-Cπ methods. Specifically, two unrelated inbreds were crossed to produce an F_1_ population, from which 2000 doubled haploid (DH) lines were generated, based on which all DH lines were mated randomly to generate 500 lines as the base population. The base population was randomly mated for 100 generations to finally form an association panel of 300 lines. The marker data were coded as *z_ij_* = 1, if *j*th marker locus in *i*th individual was homozygous for marker allele from parental Inbred 1, *z_ij_* = -1 if homozygous for marker allele from parental Inbred 2 and 0 if heterozygous. No structure effect was simulated in the study.

The genome model for simulation was constructed according to the published maize ISU–IBM genetic map, with a total of 1788 cM [[5](#_ENREF_5)], with recombination computed using the Kosambi map function [[6](#_ENREF_6)]. One hundred QTLs were positioned across the genome with average of 10 QTLs per one chromosome. Markers were evenly spaced on the chromosome at 1cM interval. Both QTLs and markers were assumed to be bi-allelic. The genotypic value for *i*th individual was calculated as the sum of additive effects:

$G_{i}=\sum_{k=1}^{L} a_{k}u_{ik}$,

Design element $u_{k}$ is defined as $u_{k}=\left\{\begin{matrix} 1 & QQ \\ 0 & Qq \\ -1 & qq \end{matrix}\right.,\text{ }$and parameter $a_{k}$is *k*th QTL’s additive effect. Additive effect$a_{k}$was sampled from a normal distribution with null mean and an empirical variance of 0.144 which was estimated from a meta-analysis using historical QTL mapping results. The genetic variance was, therefore, calculated from the sample variance of genotypic values. Markers with minor allele frequency (*i.e*. smaller than 0.1) were filtered out before running the model. Simulations were repeated 60 times.

The power to detect QTLs for all methods was calculated as the proportion of QTLs detected by significant SNPs within certain window size (i.e. QTL support interval). The window size was set at the interval of 2.5, 5, 7.5 and 10cM from both sides of the significant SNP, which defined QTL support intervals as 5, 10, 15 and 20 cM, respectively. The false positive rate (FPR) was defined as the proportion of SNPs which were not associated with any simulated QTL within certain window size.

# results of simulation tests

## Noise reduction component of SBayes method (SBayes vs. Bayes-Cπ )

To test how efficient noise reduction component of SBayes method is in QTL detection compared to Bayes-Cπ, a simulation example was shown below. On chromosome 1, eleven QTLs with small, moderate and large effects were simulated and both SBayes and Bayes-Cπ were applied to the same simulation data (Figure 1-2). SBayes method detected four out of eleven QTL, including two with high effect (QTL19 and QTL8) and two with moderate effect (QTL57 and QTL 1) (Figure 1). While Bayes-Cπ detected only two QTLs (QTL57 and QTL19), which were in common with those detected by SBayes (Figure 2). We believe that the noise reduction component (SPCA) allowed SBayes to have much more clear detection signals than the ones produced by Bayes-Cπ (Figure 1). Meanwhile, seven out of eleven QTL were not detected by either of the models which may be due to the weak SNP-QTL associations (Figure1). Out of seven undetected QTL on chromosome 1, four (QTL85, QTL74, QTL83, and QTL43) were simulated as small effects and three (QTL95, QTL6, and QTL72) were simulated as moderate effects. The power of detecting small and moderate QTL effect may be further increased by the selection of optimal marker subset and optimal PC variation.

## Parameters controlling SBayes QTL mapping power

Across statistical methods, window size increase improved the power to detect QTL effects but also elevated the FPR (Table 1-3). Empirically, in SBayes a window size of 10 to 15cM (5 and 7.5 cM from each side of the discovered marker) would lead to relatively low FPR. Except window size, in SBayes another key factor to increase power and drop FPR is the threshold. Because SBayes fits all markers simultaneously, the method doesn’t have the multi-comparison testing problem as the single marker methodology such as QK method. Theoretically, all non-zero effect markers should be considered significant, however, relatively high FPR would be obtained if low threshold was used (Table 1-2). Tables 1-3 show that empirically the threshold of 0.7, i.e. SNPs with at least 70% of the maximum heritability across the chromosome will balance detection power and FPR. Current window size and threshold was obtained based on simulation analysis implemented with about 1800 markers. For a dataset with a larger number of markers, the optimized threshold and window size would be expected to be different.

While comparing SBayes and QK models, the former appears to be more powerful. Given the same level of FPR *i.e.* 0.35 and 15 cM window size (Table 2), the power to detect all QTL with SBayes method was 0.24 at threshold of 0.8 (Table 1), while QTL detection power with QK method (FPR = 0.35; window size = 15 cM) was 0.15 (Table 3). The lack of power shown in QK method was possibly due to the inability of handling all markers simultaneously and high level of noise in the data.


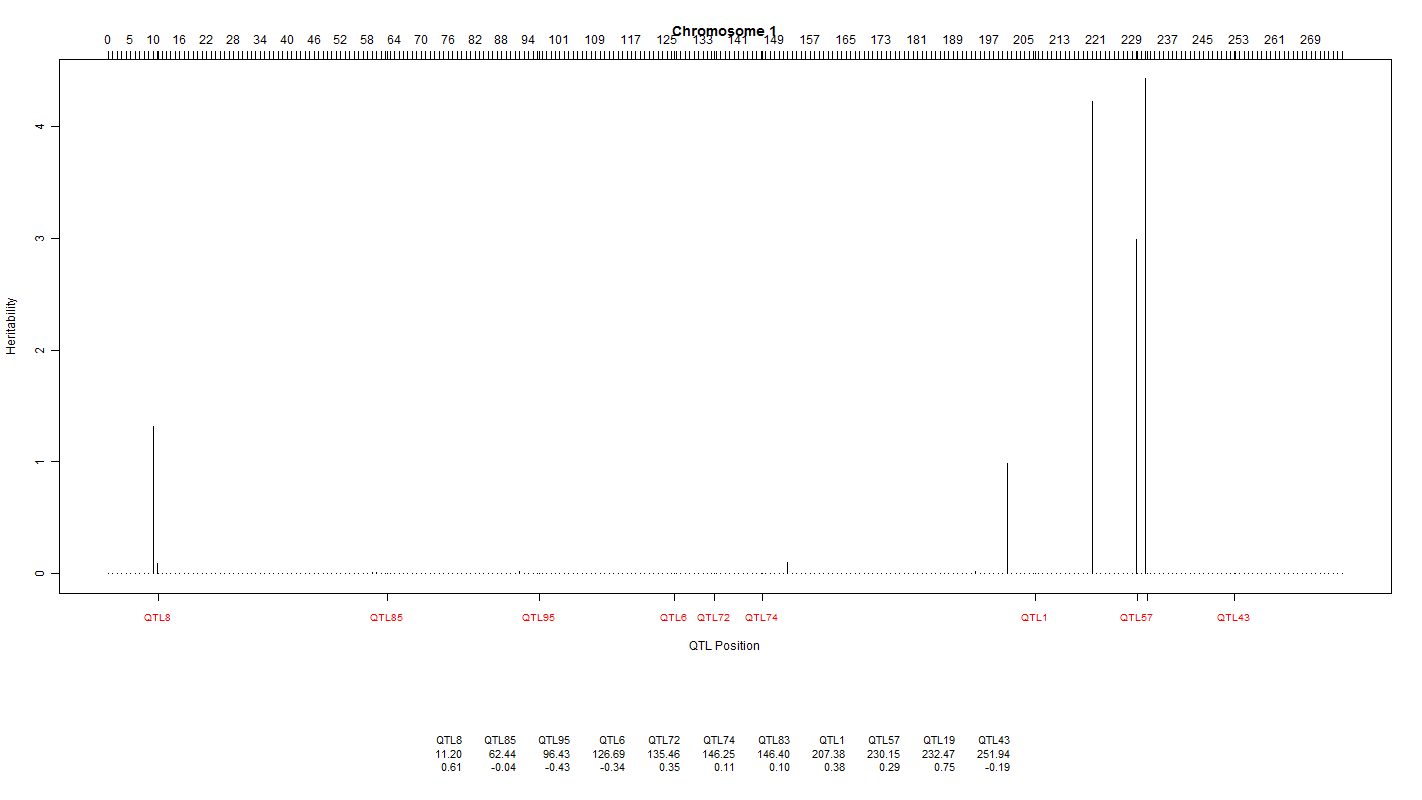


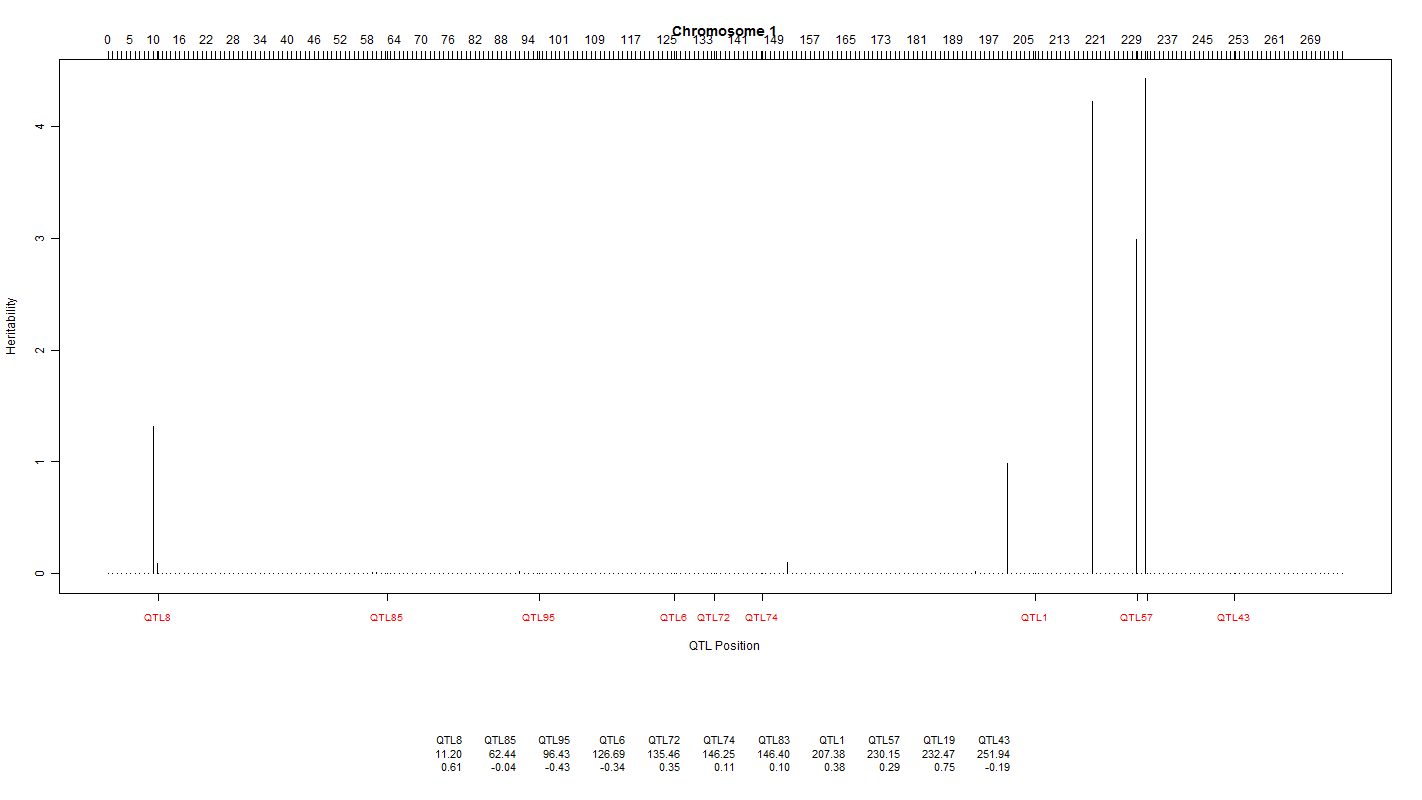


QTL 19

Figure 1. Bar plot for SNP effects in Chromosome 1 using SBayes model. X-axis represents the position of simulated QTL and y-axis represents the heritability of each SNP. Genetic distances were displayed on top x-axis and the simulated QTL genetic distances, additive effects were shown in the table at the bottom with discovered QTLs highlighted. Newly discovered QTLs by SBayes were highlighted in the bar plot.

**
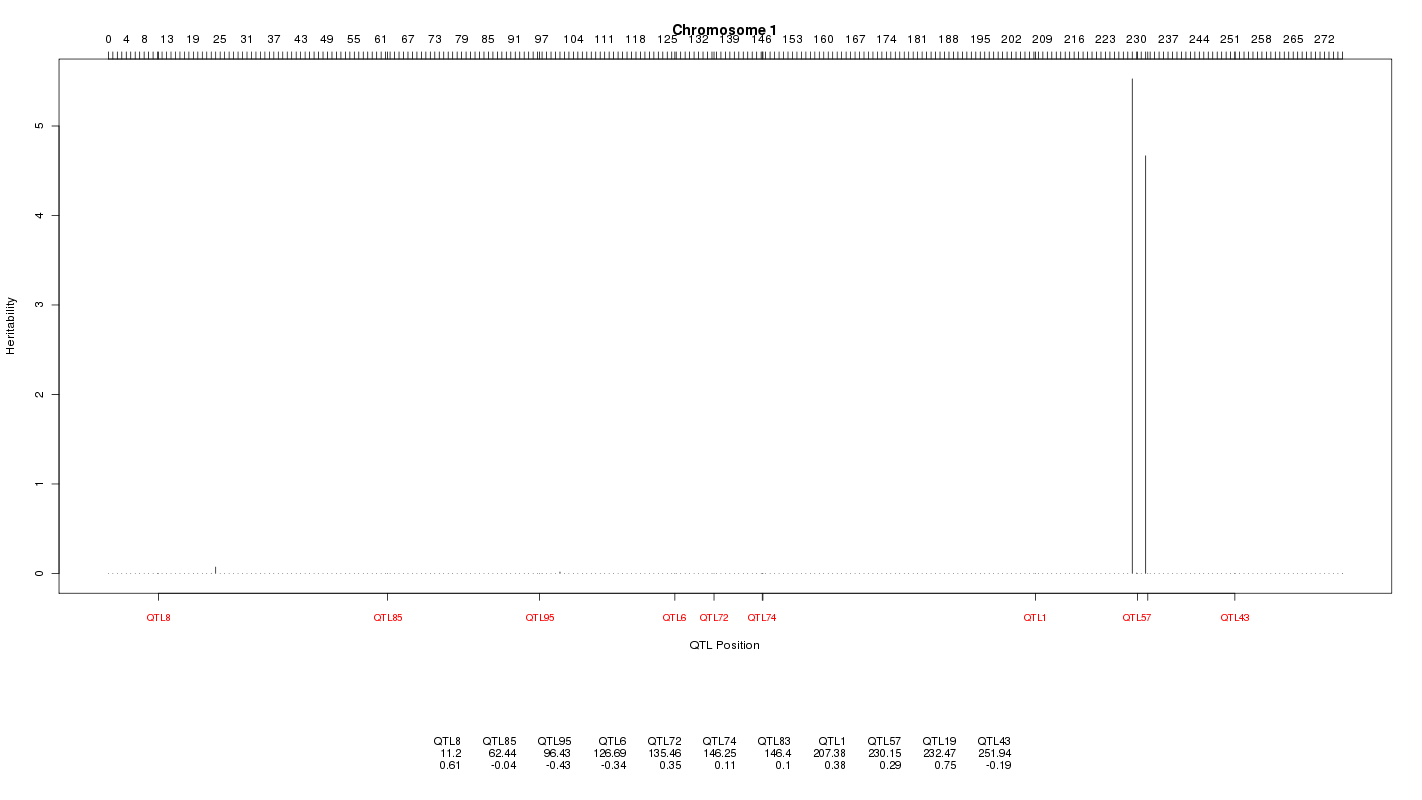

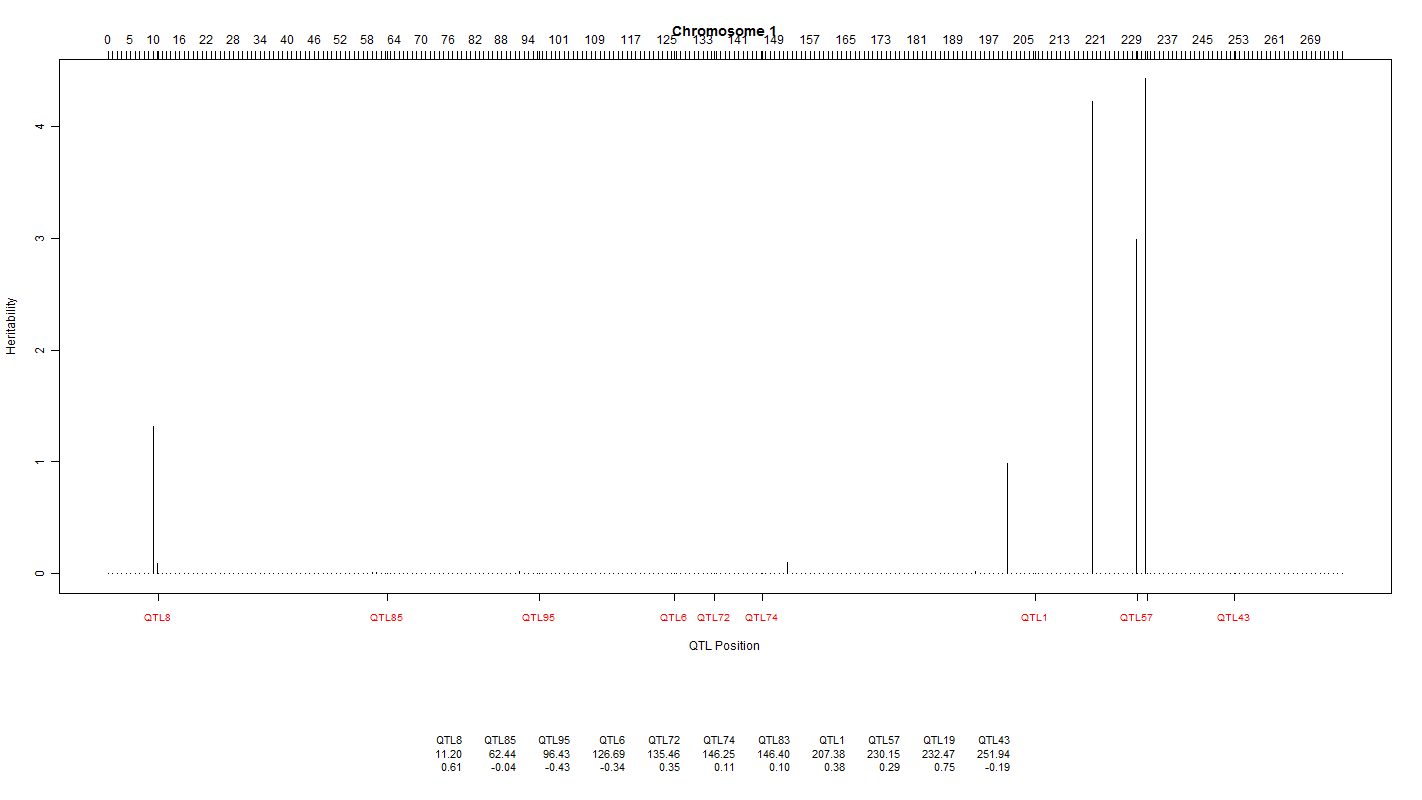
**

QTL 19

#

Figure 2. Bar plot for SNP effects in Chromosome 1 using SBayes model. X-axis represents the position of simulated QTL and y-axis represents the heritability of each SNP. Genetic distances were displayed on top x-axis and the simulated QTL genetic distances, additive effects were shown in the table at the bottom with discovered QTLs highlighted. Newly discovered QTLs by SBayes were highlighted in the bar plot.

Table 1 Mean ± sd of the estimated power to detect all effects across 60 simulations using SBayes model. The values were obtained for 24 scenarios (four types of window sizes × six types of thresholds). Window size is represented as a support interval spanning from both side of a QTL.

|  | Window size | | | |
| --- | --- | --- | --- | --- |
| Threshold (% of h^2^) | 5cM | 10cM | 15cM | 20cM |
|  | Power to detect QTL | | | |
| 0.3 | 0.29±0.07 | 0.37±0.06 | 0.40±0.09 | 0.42±0.10 |
| 0.4 | 0.27±0.07 | 0.34±0.05 | 0.37±0.08 | 0.38±0.08 |
| 0.5 | 0.23±0.07 | 0.29±0.07 | 0.32±0.10 | 0.32±0.10 |
| 0.6 | 0.22±0.08 | 0.27±0.07 | 0.30±0.10 | 0.30±0.10 |
| 0.7 | 0.20±0.07 | 0.24±0.05 | 0.26±0.07 | 0.26±0.07 |
| 0.8 | 0.19±0.07 | 0.22±0.05 | 0.24±0.07 | 0.24±0.07 |

**Table 2 Mean ± sd of the estimated false positive rate across 60 simulations using SBayes model. The values were obtained for 24 scenarios including four types of window size and six types of thresholds.**

|  | Window size | | | |
| --- | --- | --- | --- | --- |
| Threshold (% of h^2^) | 5cM | 10cM | 15cM | 20cM |
|  | False Positive Rate | | | |
| 0.3 | 0.68±0.08 | 0.6±0.08 | 0.52±0.10 | 0.47±0.11 |
| 0.4 | 0.64±0.08 | 0.57±0.07 | 0.48±0.09 | 0.41±0.10 |
| 0.5 | 0.65±0.09 | 0.58±0.09 | 0.49±0.13 | 0.41±0.14 |
| 0.6 | 0.64±0.14 | 0.55±0.11 | 0.45±0.16 | 0.39±0.16 |
| 0.7 | 0.59±0.13 | 0.48±0.09 | 0.37±0.13 | 0.33±0.13 |
| 0.8 | 0.57±0.11 | 0.45±0.08 | 0.35±0.13 | 0.32±0.13 |

**Table 3 Mean ± sd of the estimated power to detect all effects across 60 simulations using traditional QK method. The values were obtained for 32 scenarios including four types of window size and eight types of False Positive Rate (FPR).**

|  | Window size | | | |
| --- | --- | --- | --- | --- |
| FPR | 5cM | 10cM | 15cM | 20cM |
|  | Power to detect QTL | | | |
| 0.05 | 0.06±0.04 | 0.06±0.04 | 0.06±0.04 | 0.06±0.04 |
| 0.1 | 0.08±0.05 | 0.08±0.05 | 0.08±0.05 | 0.08±0.05 |
| 0.15 | 0.09±0.05 | 0.09±0.05 | 0.09±0.05 | 0.09±0.05 |
| 0.2 | 0.10±0.05 | 0.10±0.05 | 0.11±0.06 | 0.11±0.06 |
| 0.25 | 0.12±0.05 | 0.12±0.05 | 0.13±0.06 | 0.13±0.06 |
| 0.3 | 0.13±0.06 | 0.13±0.06 | 0.14±0.07 | 0.14±0.07 |
| 0.35 | 0.14±0.07 | 0.14±0.07 | 0.15±0.08 | 0.16±0.08 |
| 0.4 | 0.15±0.07 | 0.16±0.08 | 0.17±0.08 | 0.17±0.08 |

# References

1. Bair E, Hastie T, Paul D, Tibshirani R: **Prediction by supervised principal components**. *J Am Stat Assoc* 2006, **101**(473):119-137.

2. Gu C: **Smoothing spline ANOVA models.** New York: Springer-Verlag; 2002.

3. Habier D, Fernando RL, Kizilkaya K, Garrick DJ: **Extension of the Bayesian alphabet for genomic selection**. *BMC bioinformatics* 2011, **12**(1):186.

4. Wang C, Rutledge J, Gianola D: **Marginal inferences about variance components in a mixed linear model using Gibbs sampling**. *Genetics Selection Evolution* 1993, **25**(1):41-62.

5. Fu Y, Wen T-J, Ronin YI, Chen HD, Guo L, Mester DI, Yang Y, Lee M, Korol AB, Ashlock DA *et al*: **Genetic dissection of intermated recombinant inbred lines using a new genetic map of maize**. *Genetics* 2006, **174**(3):1671-1683.

6. Kosambi DD: **The estimation of map distance from recombination values**. *Annals of Eugenics* 1944, **12**(3):172-175.

7. Yu J, Pressoir G, Briggs WH, Vroh Bi I, Yamasaki M, Doebley JF, McMullen MD, Gaut BS, Nielsen DM, Holland JB *et al*: **A unified mixed-model method for association mapping that accounts for multiple levels of relatedness**. *Nat Genet* 2006, **38**(2):203-208.

8. Hayes BJ, Goddard ME: **Technical note: Prediction of breeding values using marker-derived relationship matrices**. *J Anim Sci* 2008, **86**(9):2089-2092.
