# Supplementary material for: Combining powers of linkage and association mapping for precise dissection of QTL controlling resistance to gray leaf spot disease in maize (Zea mays L.)
Source: BMC Genomics. 2015 Nov 10;16:916. doi: 10.1186/s12864-015-2171-3 (PMC4641357; doi:10.1186/s12864-015-2171-3)
Supplement: Additional file 4: — Analysis of the population structure within the set of 300 maize inbred lines used for the GWAS study. Estimated LnP(D) and Delta K averaged over five repeats of STRUCTURE analysis. (DOCX 67 kb) [file 12864_2015_2171_MOESM4_ESM.docx]

Additional file 4. **Analysis of the population structure within the set of 300 maize inbred lines used for the GWAS study.** Estimated LnP(D) and Delta K averaged over five repeats of STRUCTURE analysis.
