# Supplementary material for: Combining powers of linkage and association mapping for precise dissection of QTL controlling resistance to gray leaf spot disease in maize (Zea mays L.)
Source: BMC Genomics. 2015 Nov 10;16:916. doi: 10.1186/s12864-015-2171-3 (PMC4641357; doi:10.1186/s12864-015-2171-3)
Supplement: Additional file 5: — Phylogenetic tree of maize accessions in Association Panel. Neighbor-joining tree of 300 maize accessions representing Association Panel. Three subgroups (stiff stalk, non-stiff stalk, and tropical germplasm) identified from the tree were color-coded. (PPTX 108 kb) [file 12864_2015_2171_MOESM5_ESM.pptx]

## Slide 1
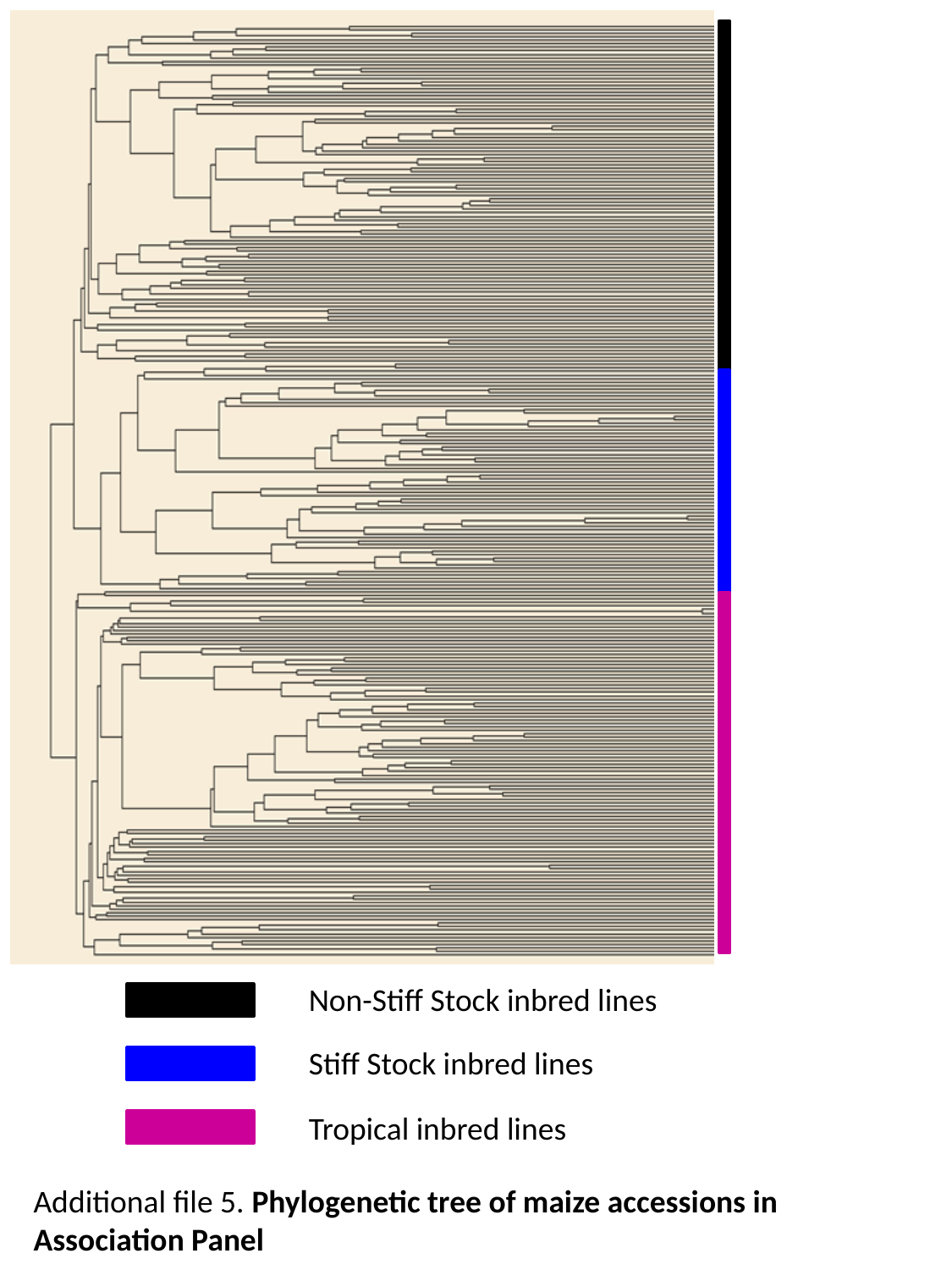

Non-Stiff Stock inbred lines
Stiff Stock inbred lines
Tropical inbred lines
Additional file 5. Phylogenetic tree of maize accessions in Association Panel
