# Supplementary material for: Combining powers of linkage and association mapping for precise dissection of QTL controlling resistance to gray leaf spot disease in maize (Zea mays L.)
Source: BMC Genomics. 2015 Nov 10;16:916. doi: 10.1186/s12864-015-2171-3 (PMC4641357; doi:10.1186/s12864-015-2171-3)
Supplement: Additional file 6: — Physical boundaries of intervals supporting GWAS-detected gray leaf spot resistance QTL. Using sequence information of GWAS-detected SNP markers associated with GLS resistance QTL and the public IBM2 2008 Neighbors map (http://www.maizegdb.org/data_center/map), corresponding chromosomal bins were identified. Physical and genetic lengths of a chromosomal bin were calculated by subtracting the physical (bp) and genetic (cM) coordinates of public markers flanking a bin where GLS resistance QTL resided. As not every flanking marker had a physical position in the public map, the closest marker with known physical positions was designated as the flanking marker. Support intervals for GWAS detected QTL were identified to be 7.5 cM from each side of a marker. Physical length of a QTL support interval = (physical length of a bin) /(genetic length of a bin)*7.5 cM. Physical boundaries of a QTL support interval = physical position of a SNP marker associated with GWAS detected QTL ± physical length of a QTL support interval. (DOCX 22 kb) [file 12864_2015_2171_MOESM6_ESM.docx]

**Additional file 6. Physical boundaries of intervals supporting GWAS-detected gray leaf spot resistance QTL**

| QTL | Bin | Flanking markers  ^a^ | Physical borders of a bin ^b^ | Approximate physical length of a bin, bp | Genetic length of a bin, cM ^c^ | Physical length of 1 cM, bp | QTL support interval, cM | QTL support interval, bp | Physical position of a maker associated with QTL, bp | Physical boundaries of support interval for a QTL |
| --- | --- | --- | --- | --- | --- | --- | --- | --- | --- | --- |
| *QTLGLSchr1* | 1.08 | AY110159 - cdj2 | 228,563,426 - 250,092,832 | 21,529,406 | 90.00 | 239,215 | 7.5 | 1,794,113 | 234,309,200 | 232,515,087 - 236,103,313 |
| *QTLGLSchr6* | 6.04 | umc65a - IDP9143 | 104,450,293 - 118,790,325 | 14,340,032 | 58.54 | 244,961 | 7.5 | 1,837,208 | 107,475,954 | 105,638,746 - 109,323,162 |
| *QTLGLSchr7* | 7.02 | IDP9113 - umc1713 | 13,615,432 - 129,865,901 | 116,250,469 | 167.94 | 692,214 | 7.5 | 5,191,605 | 19,500,572 | 14,308,967 - 24,692,177 |
| *QTLGLSchr8a* | 8.02 | mmp57 - umc1530 | 10,866,879 – 22,245,756 | 11,378,877 | 63.1 | 180,331 | 7.5 | 1,352,481 | 19,550,800 | 18,198,319 - 20,903,281 |
|  |  |  |  |  |  |  |  |  | 21,753,432 | 20,400,951 - 23,105,913 |
| *QTLGLSchr8b* | 8.03 | umc1530 - IDP8545 | 22,245,756 - 105,568,422 | 83,322,666 | 118.56 | 702,789 | 7.5 | 5,270,918 | 79,142,282 | 73,871,364 - 84,413,200 |
|  |  |  |  |  |  |  |  |  | 87,682,262 | 82,411,344 - 92,953,180 |

^a,b,c^ Flanking markers and their genetic and physical positions were retrieved from the public map high resolution IBM2 2008 Neighbors map at Maize GDB (http://www.maizegdb.org/data_center/map).
